# China’s biodiversity hotspots revisited: A treasure chest for plants

**DOI:** 10.3897/phytokeys.130.38417

**Published:** 2019-08-29

**Authors:** Jie Cai, Wen-Bin Yu, Ting Zhang, Hong Wang, De-Zhu Li

**Affiliations:** 1 Germplasm Bank of Wild Species, Kunming Institute of Botany, Chinese Academy of Sciences, Kunming, Yunnan 650201, China; 2 Center for Integrative Conservation, Xishuangbanna Tropical Botanical Garden, Chinese Academy of Sciences, Mengla, Yunnan 666303, China; 3 Key Laboratory for Plant Diversity and Biogeography of East Asia, Kunming Institute of Botany, Chinese Academy of Sciences, Kunming, Yunnan 650201, China; 4 Southeast Asia Biodiversity Research Institute, Chinese Academy of Science, Yezin, Nay Pyi Taw 05282, Myanmar; 5 Center of Conservation Biology, Core Botanical Gardens, Chinese Academy of Sciences, Mengla, Yunnan 666303, China

## Abstract

n/a

China has been recognised as having exceptionally high plant biodiversity since the mid-19^th^ century, when western plant explorers brought their discoveries to the attention of modern botany (Bretschneider 1898). The "Flora of China" recorded 31,362 vascular plant species, half of which could not be found anywhere else on earth (Raven and Hong 2013), making China one of the planet’s biologically wealthiest countries.

Biodiversity hotspots, by definition, are areas with exceptional concentrations of endemic species (containing at least 0.5% of the Earth’s plant species as endemics) and are experiencing increasing, large-scale habitat loss, at least 70% of which is caused by human disturbance (Myers et al. 2000). China hosts, mostly or partially, four of the world’s 36 biodiversity hotspots (Myers et al. 2000, CEPF 2019). The hotspots in China range from the arid northwest of the country, across Qinghai-Tibet Plateau, the highest and largest plateau of the world, to the tropical and subtropical southern China. The "Mountains of Central Asia" biodiversity hotspot reaches its eastern limit in China, including the eastern Tien Shan Mountains in central Xinjiang and the mountain ranges along China-Kyrgyzstan and China-Tajikistan borders. The mountains that surround the southern part of the Qinghai-Tibet Plateau, which extend from the western barrens to the humid southeast in Tibet (Xizang), form a significant portion of the "Himalaya" biodiversity hotspot. The "Mountains of Southwest China" biodiversity hotspot is found almost entirely within China, stretching from southeast Tibet, through western Sichuan and extending into northwest Yunnan, with only a narrow range along the western slope of the Gaoligong Mountains, located in northern Myanmar. The northeast part of the "Indo-Burma" biodiversity hotspot begins in the west of Yunnan, crosses over southern Yunnan to central Guangxi and then runs along the coast of southern China from the Guangxi-Vietnam border to eastern Guangdong, including the entire Hainan Island.

Biodiversity hotspots play a substantial role in understanding China’s unique flora. Currently, the native vascular flora from China’s biodiversity hotspots has yet to be investigated in its entirety and the estimated number of species could be more than 25,000 based on the "Flora of China" and recent surveys (The Biodiversity Committee of Chinese Academy of Sciences 2019). This accounts for approximately three quarters of China’s flora. China’s biodiversity hotspots are mainly located in remote mountain areas, where access is difficult and there are diverse microclimates. These isolated habitats are often associated with high levels of endemism and there is great potential for the discovery of new plant species in these regions (Joppa et al. 2011). Although rapid economic growth and urbanisation in China is driving landscape modification and environmental deterioration, the flourishing development of infrastructure, such as expansion of road networks, has improved accessibility to remote areas, thereby fostering the discovery of additional undescribed diversity in the country. For example, according to records in the International Plant Names Index (IPNI), 1038 new vascular plant species were described or reported for China from 2013 (the year when the "Flora of China" was completed) to the end of 2018, with some 73% of the species deriving from China’s biodiversity hotspots (Figure 1, Appendix 1). The future discovery of additional new plant species in these regions is likely.

**Figure 1.**
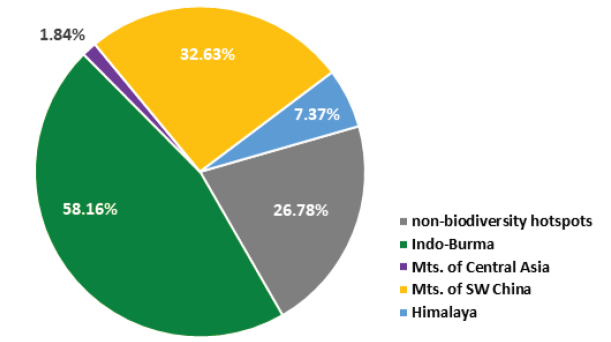
Chart of new species reported to occur in China’s biodiversity hotspots, based on data from IPNI, 1 Jan. 2013- 31 Dec. 2018

The "Flora of China" represents the most comprehensive catalogue, description and illustration of known vascular plant species of China. However, a few of the early treatments published were essentially updated translations of the "Flora Reipublicae Popularis Sinicae" and for a number of other groups, no specialists were available at the time when the treatments were completed, so that they are in essence preliminary. A great deal of further taxonomic work, often simulated by the "Flora", has improved our understanding of many groups, very often adding to the number of recognised species. For example, recent taxonomic revisions of Chinese *Aristolochia*, which included only 45 species in the "Flora of China", split the genus into two genera with 78 species in total, of which 19 were newly described (Zhu et al. 2019b, Zhu X.-X. personal communication), two of them in this special issue (Zhu et al. 2019a). It is quite clear than many more species are present in China than are now recognized, and likely that most of these will come from the hotspots.

The application of DNA barcodes and other sequence data in the past decade has helped to improve our understanding of the species and relationships in many groups of plants (Kress et al. 2005, CBOL Plant Working Group 2009, China Plant BOL Group 2011, Hollingsworth et al. 2016). To fully understand the diverse flora of China, these tools must be applied widely (e.g. Liu et al. 2011, Yan et al. 2015, Yu et al. 2015).

To better document, understand and conserve China’s biological heritage, Chinese scientists have conducted a series of initiatives to facilitate the understanding and conservation of plant diversity, particularly over the past twenty years. These range from baseline, floristic surveys to long-term monitoring studies that document dynamic patterns of biodiversity with a specific focus on Southwest China and the Qinghai-Tibet Plateau, where many ecosystems are being degraded as a result of human activities and global warming (Liu et al. 2018). Indeed, much action is urgently needed to mitigate the effect of human disturbance and climate change in these hotspots and elsewhere in China.

With extended collaboration amongst Chinese scientists and coordination of networks on plant conservation and taxonomy across China, we have synthesised a special issue entitled “Revealing the plant diversity in China’s biodiversity hotspots”, to present the latest findings by Chinese botanists and to update knowledge of the flora for China and adjacent countries. This issue, comprising 18 articles, includes descriptions of 23 species new to science and new insights into the diversity of *Scleroglossum* (Polypodiaceae) based on DNA barcodes. The new species originate from the following hotspots: Indo-Burma (13), Mountains of Southwest China (2), Himalaya (4), Mountains of Central Asia (1) and 3 from areas with conservation interest outside the hotspots.

The new species published in this special issue reflect ongoing taxonomic and floristic research across China. It is hoped that these new discoveries will contribute to the objectives of the updated Global Strategy for Plant Conservation (GSPC) 2011–2020 and China’s Plant Conservation Strategy, in general. In particular, we also strive to facilitate national and local policy-makers to develop more effective conservation guidelines. The naming and describing of new species are fundamental steps for understanding China’s natural history and assessing its plant diversity is the first “stepping-stone” to securing the success of our biodiversity initiative. In short, the value of botanical inventories and taxonomic work should be recognised through prioritised funding opportunities, inspired taxonomy training schemes, wider public involvement and an integrated GSPC post-2020 framework.

## Appendix 1

List of new vascular plant species occured or described from China between 2013–2018 (data from IPNI).

**Table:**

Family	Scientific name	Publication year	Discovered from Biodiversity hotspots*	IPNI Plant Name Id
Acanthaceae	*Justicia weihongjinii* Y.F.Deng, Y.Tong & Y.S.Huang	2016	IB	77153239-1
	*Rungia flaviflora* Z.L.Lin & Y.F.Deng	2018	IB	77185931-1
	*Rungia sinothailandica* Z.L.Lin & Y.F.Deng	2017	IB	77174700-1
Adiantaceae	*Adiantum × ailaoshanense* Y.H.Yan & Ying Wang	2015	IB	77151658-1
	*Adiantum dentatum* A.H.Wang, F.G.Wang & F.W.Xing	2017	IB	77160197-1
	*Adiantum longzhouensis* A.H.Wang, F.G.Wang & F.W.Xing	2017	IB	77160199-1
	*Adiantum obovatum* A.H.Wang, F.G.Wang & F.W.Xing	2017	IB	77160198-1
	*Coniogramme bashanensis* X.S.Guo & Bin Li	2013		77129783-1
Alangiaceae	*Alangium indochinense* W.J.de Wilde & Duyfjes	2016	IB	77160208-1
Alliaceae	*Allium kirilovii* N.Friesen & Seregin	2015	CA	77147239-1
	*Allium montanostepposum* N.Friesen & Seregin	2015		77147238-1
Amaryllidaceae	*Lycoris × hubeiensis* Kun Liu	2018		77188002-1
	*Lycoris hunanensis* M.H.Quan, L.J.Ou & C.W.She	2013		77132590-1
Annonaceae	*Alphonsea glandulosa* Y.H.Tan & B.Xue	2017	IB	77159533-1
	*Polyalthia yingjiangensis* Y.H.Tan & B.Xue	2017	IB	77174697-1
Apiaceae	*Acronema crassifolium* Huan C.Wang, X.M.Zhou & Y.H.Wang	2013	SW	77127272-1
	*Angelica muliensis* C.Y.Liao & X.G.Ma	2017	SW	77179082-1
	*Bupleurum baimaense* X.G.Ma & X.J.He	2013	SW	77135889-1
	*Bupleurum dracaenoides* Huan C.Wang, Z.R.He & H.Sun	2014	SW	77136465-1
	*Bupleurum shanianum* X.G.Ma & X.J.He	2014	SW	77146546-1
	*Chamaesium jiulongense* X.L.Guo & X.J.He	2017	SW	77174717-1
	*Hydrocotyle peltiformis* R.Li & H.Li	2013	SW	77144604-1
	*Libanotis laoshanensis* W.Zhou & Q.X.Liu	2015		77154495-1
	*Ostericum atropurpureum* G.Y.Li, G.H.Xia & W.Y.Xie	2013		77131197-1
	*Pternopetalum monophyllum* J.B.Tan & X.J.He	2014	SW	77145115-1
	*Pternopetalum porphyronotum* J.B.Tan	2018	SW	77179091-1
	*Semenovia gyirongensis* Q.Y.Xiao & X.J.He	2017	H	77163815-1
Aquifoliaceae	*Ilex calcicola* W.B.Liao & K.W.Xu	2017	IB	77177671-1
	*Ilex chuguangii* M.M.Lin	2013	IB	77129105-1
	*Ilex gansuensis* D.Y.Hong	2015		77150544-1
	*Ilex jingxiensis* Y.F.Huang & M.X.Lai	2014	IB	77144567-1
	*Ilex sanqingshanensis* W.B.Liao, Q.Fan & S.Shi	2015		77154790-1
	*Ilex shukunii* Y.Yang & H.Peng	2018	SW	77192550-1
	*Ilex venusta* H.Peng & W.B.Liao	2017		77161288-1
Araceae	*Amorphophallus bubenensis* J.T.Yin & Hett.	2016	IB	77157352-1
	*Arisaema chenii* Z.X.Ma & Yi Jun Huang	2018	IB	77195372-1
	*Arisaema longitubum* Z.X.Ma	2018	SW	77186325-1
	*Pinellia hunanensis* C.L.Long & X.J.Wu	2013		77133084-1
Arecaceae	*Guihaia lancifolia* K.W.Luo & F.W.Xing	2017	IB	77161298-1
Aristolochiaceae	*Aristolochia compressicaulis* Z.L.Yang	2015	SW	60469243-2
	*Aristolochia gongchengensis* Y.S.Huang, Y.D.Peng & C.R.Lin	2015	IB	77174904-1
	*Aristolochia huanjiangensis* Yan Liu & L.Wu	2013	IB	77135912-1
	*Aristolochia hyperxantha* X.X.Zhu & J.S.Ma	2017		77176269-1
	*Aristolochia involuta* X.X.Zhu, Z.X.Ma & J.S.Ma	2017	IB	77174882-1
	*Aristolochia longlinensis* Yan Liu & L.Wu	2015	IB	77148099-1
	*Aristolochia melanocephala* X.X.Zhu & J.S.Ma	2018	IB	77192596-1
	*Aristolochia mulunensis* Y.S.Huang & Yan Liu	2013	IB	77130236-1
	*Aristolochia pilosistyla* X.X.Zhu & J.S.Ma	2018	IB	77192597-1
	*Aristolochia pseudocaulialata* X.X.Zhu, J.N.Liu & J.S.Ma	2018	IB	77187811-1
Aristolochiaceae	*Aristolochia sinoburmanica* Y.H.Tan & B.Yang	2018	IB	60475913-2
	*Aristolochia tongbiguanensis* J.Y.Shen, Q.B.Gong & Landrein	2018	IB	77190831-1
	*Aristolochia weixiensis* X.X.Zhu & J.S.Ma	2015	SW	77150406-1
Asclepiadaceae	*Hoya mcclurei* Kloppenb.	2018	IB	60476784-2
	*Hoya yingjiangensis* J.Feng Zhang, L.Bai, N.H.Xia & Z.Q.Peng	2015	IB	77148268-1
	*Vincetoxicum xinpingense* H.Peng & Y.H.Wang	2018	IB	77187228-1
Aspleniaceae	*Asplenium × huawuense* Z.R.Wang ex Viane & Y.X.Lin	2013		77133567-1
	*Asplenium × kidoi* Sleep ex Viane, Y.X.Lin & Reichst.	2013		77133568-1
	*Asplenium × mickelii* Viane & Reichst.	2013		77133561-1
	*Asplenium × mitsutae* Viane & Reichst.	2013	SW	77133563-1
	*Asplenium × wudangshanense* Viane, Reichst., Rasbach & Y.X.Lin	2013		77133564-1
	*Asplenium cyrtosorum* K.W.Xu, Li Bing Zhang & W.B.Liao	2018	IB	77179208-1
	*Asplenium guangdongense* Y.Fen Chang & H.Schneid.	2018	IB	77190779-1
	*Asplenium mae* Viane & Reichst.	2013		77133482-1
	*Asplenium normaloides* Y.Fen Chang & H.Schneid.	2018	IB	77190778-1
	*Hymenasplenium chingii* K.W.Xu, Li Bing Zhang & W.B.Liao	2018	IB	77186055-1
	*Hymenasplenium denticulatum* K.W.Xu, Li Bing Zhang & W.B.Liao	2018		77186056-1
	*Hymenasplenium hastifolium* K.W.Xu, Li Bing Zhang & W.B.Liao	2018	IB	77176507-1
	*Hymenasplenium laterepens* N.Murak. & X.Cheng ex Y.Fen Chang & K.Hori	2018	IB	77191769-1
	*Hymenasplenium pseudobscurum* Viane	2013	IB	77133491-1
	*Hymenasplenium sinense* K.W.Xu, Li Bing Zhang & W.B.Liao	2018		77186062-1
	*Hymenasplenium speluncicola* Li Bing Zhang, K.W.Xu & H.He	2018	IB	77186063-1
	*Hymenasplenium wangpeishanii* Li Bing Zhang & K.W.Xu	2018		77186064-1
Asteraceae	*Anaphalis hymenolepis* Y.Ling	2013	SW	77133458-1
	*Aster oliganthus* W.P.Li & Zhi Li	2017	SW	77177658-1
	*Aster tianmenshanensis* G.J.Zhang & T.G.Gao	2015		77148277-1
	*Aster veitchianus* Hutch. & J.R.Drumm. ex G.J.Zhang & T.G.Gao	2013	SW	77136165-1
	*Chrysanthemum yantaiense* M.Sun & J.T.Chen	2018		77191578-1
	*Chrysanthemum zhuozishanense* L.Q.Zhao & Jie Yang	2014		77147571-1
	*Cremanthodium bomiense* L.Wang, C.Ren & Q.E.Yang	2016	H	77159886-1
	*Cremanthodium hongshanense* L.Wang, C.Ren & Q.E.Yang	2018	SW	77187042-1
	*Cremanthodium liangshanicum* L.Wang, C.Ren & Q.E.Yang	2016	SW	77159851-1
	*Cremanthodium maoxianense* L.Wang, C.Ren & Q.E.Yang	2018	SW	77187043-1
	*Cremanthodium wumengshanicum* L.Wang, C.Ren & Q.E.Yang	2015		77151705-1
	*Faberia pinnatifida* Ying Liu, Y.S.Chen & Boufford	2018	SW	77185933-1
	*Hieracium sinoaestivum* Sennikov	2014		77140258-1
	*Ligularia jiajinshanensis* Y.S.Chen	2016	SW	77159887-1
	*Ligularia lhunzensis* Y.S.Chen	2016	H	77159888-1
	*Ligularia secunda* Y.S.Chen	2016	H	77159889-1
	*Ligularia zhengyiana* Xin W.Li, Q.Luo & Q.L.Gan	2014		77144564-1
	*Melanoseris jilongensis* Ze H.Wang & H.Peng	2018	H	77185970-1
	*Parasenecio anhuiensis* Y.S.Chen & Lian S.Xu	2016		77159301-1
	*Pertya markamensis* Cai F.Zhang & T.G.Gao	2017	SW	77165473-1
	*Pertya multiflora* Cai F.Zhang & T.G.Gao	2013		77133645-1
	*Saussurea austrotibetica* Y.S.Chen	2014	H	77143044-1
	*Saussurea bhutanensis* Y.S.Chen	2014	H	77143048-1
	*Saussurea bijiangensis* Y.L.Chen ex B.Q.Xu, N.H.Xia & G.Hao	2013	SW	77131020-1
	*Saussurea chinduensis* Y.S.Chen	2015		77147918-1
	*Saussurea dulongjiangensis* Y.S.Chen	2015	SW	77147919-1
	*Saussurea fuscipappa* Y.S.Chen	2014	H	77140926-1
	*Saussurea glandulosissima* Raab-Straube	2017	H	77161042-1
	*Saussurea gongriensis* Y.S.Chen	2015	H	77147909-1
	*Saussurea habashanensis* Y.S.Chen	2015	SW	77147906-1
	*Saussurea haizishanensis* B.Q.Xu, G.Hao & N.H.Xia	2013	SW	77138680-1
	*Saussurea hengduanshanensis* Raab-Straube	2017	H	77161044-1
Asteraceae	*Saussurea jindongensis* Y.S.Chen	2015	H	77147905-1
	*Saussurea jiulongensis* Y.S.Chen	2015	SW	77147925-1
	*Saussurea kawakarpo* Raab-Straube	2017	SW	77161043-1
	*Saussurea langpoensis* Y.S.Chen	2014	H	77143045-1
	*Saussurea lhozhagensis* Y.S.Chen	2014	H	77143049-1
	*Saussurea lhunzensis* Y.S.Chen	2014	H	77143046-1
	*Saussurea liangshanensis* Y.S.Chen	2014	SW	77140928-1
	*Saussurea minutiloba* Y.S.Chen	2015	SW	77147910-1
	*Saussurea multiloba* Y.S.Chen	2015	SW	77147911-1
	*Saussurea nyingchiensis* Y.S.Chen	2015	H	77147915-1
	*Saussurea pagriensis* Y.S.Chen	2014	H	77143047-1
	*Saussurea pseudoeriostemon* Y.S.Chen	2015	H	77147912-1
	*Saussurea pseudograminea* Y.F.Wang, G.Z.Du & Y.S.Lian	2014	SW	77138689-1
	*Saussurea pseudojiulongensis* Y.S.Chen	2015	SW	77147928-1
	*Saussurea pseudoleucoma* Y.S.Chen	2015	H	77147902-1
	*Saussurea pseudolingulata* Y.S.Chen	2015	SW	77147913-1
	*Saussurea pseudoplatyphyllaria* Y.S.Chen	2015	SW	77147907-1
	*Saussurea pseudorockii* Y.S.Chen	2014	SW	77140929-1
	*Saussurea pseudosimpsoniana* Y.S.Chen	2015	H	77147903-1
	*Saussurea pseudotridactyla* Y.S.Chen	2015	H	77147901-1
	*Saussurea pseudoyunnanensis* Y.S.Chen	2015	SW	77147920-1
	*Saussurea qamdoensis* Y.S.Chen	2014	H	77140930-1
	*Saussurea septentrionalis* Raab-Straube	2017	SW	77161045-1
	*Saussurea shangrilaensis* Y.S.Chen	2014	SW	77141455-1
	*Saussurea shuiluoensis* Y.S.Chen	2015	SW	77147924-1
	*Saussurea sichuanica* Raab-Straube	2017	SW	77161046-1
	*Saussurea sikkimensis* Raab-Straube	2017		77161041-1
	*Saussurea sobarocephaloides* Y.S.Chen	2015	SW	77147923-1
	*Saussurea sunhangii* Raab-Straube	2017	SW	77161049-1
	*Saussurea tsoongii* Y.S.Chen	2015		77147900-1
	*Saussurea wenchengiae* B.Q.Xu, G.Hao & N.H.Xia	2013		77130903-1
	*Saussurea xianrendongensis* Y.S.Chen	2015	SW	77147914-1
	*Saussurea xiaojinensis* Y.S.Chen	2014	SW	77140931-1
	*Saussurea yangii* Y.S.Chen	2015	SW	77147929-1
	*Saussurea yanyuanensis* Y.S.Chen	2015	SW	77147921-1
	*Saussurea yui* Y.S.Chen	2015	SW	77147908-1
	*Saussurea zayuensis* Y.S.Chen	2015	H	77147916-1
	*Saussurea zogangensis* Y.S.Chen	2015		77147904-1
	*Senecio changii* C.Ren & Q.E.Yang	2016	SW	77153507-1
	*Senecio pseudodensiserratus* T.J.Tong, M.Tang, C.Ren & Q.E.Yang	2018	SW	77178965-1
	*Sphagneticola × guangdongensis* Q.Yuan	2015	IB	60469219-2
	*Youngia baoxingensis* Y.S.Chen	2018	SW	77192561-1
	*Youngia gongshanensis* Y.S.Chen & R.Ke	2016	SW	77157759-1
	*Youngia jiulongensis* Y.L.Peng, X.F.Gao & Li Bing Zhang	2017	SW	77167357-1
	*Youngia purpimea* Y.L.Peng, W.B.Ju, X.F.Gao & Y.D.Gao	2015	SW	77151636-1
	*Youngia zhengyiana* T.Deng, D.G.Zhang, J.W.Zhang & H.Sun	2014		77141045-1
Balsaminaceae	*Impatiens baokangensis* Q.L.Gan & X.W.Li	2016		77157482-1
	*Impatiens guiqingensis* S.X.Yu	2016	SW	77158514-1
	*Impatiens liboensis* K.M.Liu & R.P.Kuang	2013		77141790-1
	*Impatiens lixianensis* S.X.Yu	2013	SW	77130092-1
	*Impatiens lizipingensis* Q.Luo	2015	SW	77150282-1
	*Impatiens menghuochengensis* Q.Luo	2014	SW	77144565-1
	*Impatiens pandurata* Y.H.Tan & S.X.Yu	2015	IB	77156517-1
	*Impatiens pterocaulis* S.X.Yu & L.R.Zhang	2013	IB	77137129-1
	*Impatiens shennongensis* Qiang Wang & H.P.Deng	2016		77152020-1
	*Impatiens tianlinensis* S.X.Yu & L.J.Zhang	2015	IB	77150201-1
Balsaminaceae	*Impatiens unguiculata* K.M.Liu & Y.Y.Cong	2013	H	77130230-1
	*Impatiens wawuensis* Bo Ding & S.X.Yu	2016	SW	77157741-1
	*Impatiens xanthinoides* G.W.Hu	2015	IB	77150202-1
Begoniaceae	*Begonia bambusetorum* H.Q.Nguyen, Y.M.Shui & W.H.Chen	2018	IB	77175485-1
	*Begonia ehuangzhangensis* Q.L.Ding, W.Y.Zhao & W.B.Liao	2018	IB	77192653-1
	*Begonia ferox* C.I Peng & Yan Liu	2013	IB	77138236-1
	*Begonia gulongshanensis* Y.M.Shui & W.H.Chen	2018	IB	77175487-1
	*Begonia jinyunensis* C.I Peng, Bo Ding & Qian Wang	2014	SW	77142829-1
	*Begonia leipingensis* D.K.Tian, Li H.Yang & Chun Li	2016	IB	77152009-1
	*Begonia longgangensis* C.I Peng & Yan Liu	2013	IB	77138235-1
	*Begonia medogensis* Jian W.Li, Y.H.Tan & X.H.Jin	2018	H	77186060-1
	*Begonia pellionioides* Y.M.Shui & W.H.Chen	2015	IB	77151735-1
	*Begonia pulchrifolia* D.K.Tian & Ce H.Li	2015	SW	77147124-1
	*Begonia qingchengshanensis* H.Z.Li, C.I Peng & C.W.Lin	2018	SW	77178758-1
	*Begonia rhytidophylla* Y.M.Shui & W.H.Chen	2018	IB	77175490-1
	*Begonia ufoides* C.I Peng, Y.H.Qin & C.W.Lin	2017	IB	77176458-1
	*Begonia wuzhishanensis* C.I Peng, X.H.Jin & S.M.Ku	2014	IB	77142841-1
Berberidaceae	*Berberis × baoxingensis* X.H.Li	2015	SW	77150118-1
	*Berberis bowashanensis* Harber	2017	SW	77165384-1
	*Berberis brevipedicellata* Harber	2016	SW	77154636-1
	*Berberis dokerlaica* Harber	2016	SW	77154634-1
	*Berberis viridiflora* X.H.Li	2017	SW	77165327-1
	*Berberis yiliangensis* Harber	2016		77154635-1
	*Berberis zhaotongensis* Harber	2017		77165383-1
	*Epimedium jinchengshanense* Yan J.Zhang & J.Q.Li	2014	SW	77141433-1
	*Epimedium muhuangense* S.Z.He & Y.Y.Wang	2017		77176610-1
	*Epimedium tianmenshanense* T.Deng, D.G.Zhang & H.Sun	2015		77149069-1
	*Epimedium xichangense* Yan J.Zhang	2016	SW	77155713-1
	*Epimedium zhaotongense* G.W.Hu	2017		77160545-1
Betulaceae	*Betula hainanensis* J.Zeng, B.Q.Ren, J.Y.Zhu & Z.D.Chen	2014	IB	77145107-1
	*Betula skvortsovii* McAll. & Ashburner	2013	SW	77128710-1
Boraginaceae	*Bothriospermum longistylum* Q.W.Lin & Bing Liu	2017		77179093-1
	*Microula roseiflora* W.T.Yu	2016	SW	77153856-1
	*Myosotis wumengensis* L.Wei	2017		77174642-1
	*Onosma lhokaensis* Y.He & Q.R.Liu	2018	H	77188007-1
	*Sinojohnstonia ruhuaii* W.B.Liao & Lei Wang	2014		77147570-1
	*Trigonotis jiaochengensis* Q.R.Liu & R.Y.Yan	2016		77174587-1
Brassicaceae	*Aphragmus pygmaeus* Al-Shehbaz	2015	SW	77153427-1
	*Braya sichuanica* Al-Shehbaz	2014	SW	60468196-2
	*Cardamine hongdeyuana* Al-Shehbaz	2015	H	77144177-1
	*Cardamine kokaiensis* Yahara, Soejima, Kudoh, Šlenker & Marhold	2018		77187973-1
	*Cardamine kuankuoshuiense* M.T.An, Yun Lin & Y.B.Yang	2016		77155969-1
	*Cardamine pseudotrifoliolata* Al-Shehbaz	2015		77153429-1
	*Cardamine xinfenii* Al-Shehbaz	2015	SW	60470438-2
	*Draba dongchuanensis* Al-Shehbaz, J.P.Yue, T.Deng & H.L.Chen	2014		77142950-1
	*Eutrema bulbiferum* Y.Xiao & D.K.Tian	2015		77148264-1
	*Eutrema giganteum* G.Q.Hao, Al-Shehbaz & J.Quan Liu	2017	SW	77163811-1
	*Eutrema nanum* G.Q.Hao, J.Quan Liu & Al-Shehbaz	2018	SW	60477015-2
	*Eutrema racemosum* Al-Shehbaz, G.Q.Hao & J.Quan Liu	2015	SW	77149658-1
	*Eutrema tianshanense* G.Q.Hao, J.Quan Liu & Al-Shehbaz	2016	CA	77159339-1
	*Eutrema zhuxiense* Q.L.Gan & Xin W.Li	2014		77147526-1
	*Hilliella rhombea* D.D.Ma & W.Y.Xie	2018		77185950-1
	*Orychophragmus longisiliquus* Huan Hu, J.Quan Liu & Al-Shehbaz	2018		77193103-1
	*Orychophragmus zhongtiaoshanus* Huan Hu, J.Quan Liu & Al-Shehbaz	2018		77193104-1
	Solms-laubachia tianbaoshanensis H.L.Chen, Al-Shehbaz, J.P.Yue & H.Sun	2018	SW	77192259-1
Buddlejaceae	*Buddleja jinsixiaensis* R.B.Zhu	2014		77138131-1
Burmanniaceae	*Thismia gongshanensis* Hong Qing Li & Y.K.Bi	2013	SW	77129874-1
	*Thismia hongkongensis* Mar & R.M.K.Saunders	2015	IB	77145069-1
Buxaceae	*Sarcococca longipetiolata* M.Cheng	2014	IB	77142444-1
Campanulaceae	*Adenophora dawuensis* D.Y.Hong	2015	SW	77153678-1
	*Adenophora linearifolia* D.Y.Hong	2015	SW	77153682-1
	*Campanula microphylloidea* D.Y.Hong	2015		77150199-1
	*Campanula rotata* D.Y.Hong	2015		77150198-1
	*Codonopsis bomiensis* D.Y.Hong	2014	SW	77141201-1
	*Codonopsis elliptica* D.Y.Hong	2014	SW	77141206-1
	*Codonopsis gongshanica* Qiang Wang & D.Y.Hong	2014	SW	77144108-1
	*Codonopsis hemisphaerica* P.C.Tsoong ex D.Y.Hong	2014	SW	77141202-1
	*Codonopsis lixianica* D.Y.Hong	2014	SW	77141205-1
	*Cyananthus ligulosus* D.Y.Hong	2015	H	77153669-1
	*Lobelia drungjiangensis* D.Y.Hong	2015	SW	77153691-1
	*Lobelia gaoligongshanica* D.Y.Hong	2015	SW	77153690-1
	*Lobelia hongiana* Q.F.Wang & G.W.Hu	2018	IB	60475915-2
	*Pseudocodon petiolatus* D.Y.Hong & Q.Wang	2015	SW	77145980-1
Capparaceae	*Capparis longgangensis* S.L.Mo & X.S.Lee ex Y.S.Huang	2013	IB	77135584-1
Caryophyllaceae	*Silene langshanensis* L.Q.Zhao, Y.Z.Zhao & Z.M.Xin	2016		77155102-1
	*Stellaria abaensis* H.F.Xu & Z.H.Ma	2018	SW	60477672-2
	*Stellaria zhuxiensis* Q.L.Gan & Xin W.Li	2014		77139160-1
Celastraceae	*Glyptopetalum verticillatum* Q.R.Liu & S.Y.Meng	2015	IB	77151396-1
	*Salacia menglaensis* J.Y.Shen, L.C.Yan & Landrein	2018	IB	77193158-1
Chenopodiaceae	*Chenopodium perttii* Sukhor.	2014	H	77144443-1
	*Corispermum iljinii* Sukhor. & M.Zhang	2014		77141446-1
	*Corispermum nanum* Sukhor. & M.Zhang	2014		77141852-1
	*Dysphania geoffreyi* Sukhor.	2015	H	77145964-1
	*Dysphania himalaica* Uotila	2013	H	77131040-1
	*Dysphania kitiae* Uotila	2013	SW	77131041-1
	*Grubovia brevidentata* G.L.Chu	2017	CA	77174792-1
	*Grubovia mucronata* G.L.Chu	2017		77174815-1
	*Micropeplis densiflora* Z.B.Wen & G.L.Chu	2017	CA	77174817-1
	*Neobotrydium corniculatum* G.L.Chu	2017	SW	77165145-1
	*Neobotrydium corniculatum* G.L.Chu & M.L.Zhang	2016	SW	77174837-1
	*Neobotrydium longii* G.L.Chu	2017	H	77165158-1
	*Neobotrydium ornithopodum* G.L.Chu	2017	SW	77165155-1
	*Neobotrydium ornithopodum* G.L.Chu & M.L.Zhang	2016	SW	77174838-1
	*Salicornia crassispica* G.L.Chu	2017	CA	77174816-1
	*Salicornia erectispica* G.L.Chu	2017	CA	77174804-1
	*Suaeda turgida* G.L.Chu	2017	CA	77165188-1
	*Sympegma elegans* G.L.Chu	2017	SW	77174808-1
Convallariaceae	*Aspidistra australis* S.Z.He & W.F.Xu	2013		77134253-1
	*Aspidistra austroyunnanensis* G.W.Hu, Lei Cai & Q.F.Wang	2018	IB	77185868-1
	*Aspidistra chongzuoensis* C.R.Lin & Y.S.Huang	2015	IB	77147323-1
	*Aspidistra chunxiuensis* C.R.Lin & Yan Liu	2015	IB	77147208-1
	*Aspidistra cleistantha* D.X.Nong & H.Z.Lü	2018	IB	77191654-1
	*Aspidistra crassifila* Yan Liu & C.I Peng	2013	IB	77142915-1
	*Aspidistra erythrocephala* C.R.Lin & Y.Y.Liang	2016	IB	77158522-1
	*Aspidistra extrorsa* C.R.Lin & D.X.Nong	2018	IB	77178757-1
	*Aspidistra guizhouensis* S.Z.He & W.F.Xu	2015		77145935-1
	*Aspidistra leucographa* C.R.Lin & C.Y.Zou	2017		77162984-1
	*Aspidistra lingchuanensis* C.R.Lin & L.F.Guo	2015	IB	77144888-1
	*Aspidistra lingyunensis* C.R.Lin & L.F.Guo	2013	IB	77137128-1
	*Aspidistra longgangensis* C.R.Lin, Y.S.Huang & Yan Liu	2015	IB	77148872-1
	*Aspidistra longshengensis* C.R.Lin & W.B.Xu	2015	IB	77147209-1
Convallariaceae	*Aspidistra maguanensis* S.Z.He & D.H.Lv	2017	IB	77174286-1
	*Aspidistra nankunshanensis* Yan Liu & C.R.Lin	2013	IB	77131012-1
	*Aspidistra ovatifolia* Yan Liu & C.R.Lin	2014	IB	77147618-1
	*Aspidistra pingfaensis* S.Z.He & Q.W.Sun	2014		77143073-1
	*Aspidistra qijiangensis* S.Z.He & X.Y.Luo	2018	SW	77186348-1
	*Aspidistra radiata* G.W.Hu & Q.F.Wang	2016	IB	77161470-1
	*Aspidistra revoluta* Hao Zhou, S.R.Yi & Q.Gao	2016	SW	77154724-1
	*Aspidistra ronganensis* C.R.Lin, Jing Liu & W.B.Xu	2016	IB	77157342-1
	*Aspidistra sessiliflora* Aver. & Tillich	2018	SW	77192625-1
	*Aspidistra sinensis* Aver. & Tillich	2016	IB	77155594-1
	*Aspidistra stenophylla* C.R.Lin & R.C.Hu	2014	IB	77140919-1
	*Aspidistra tenuifolia* C.R.Lin & J.C.Yang	2014	IB	77138489-1
	*Aspidistra wujiangensis* W.F.Xu & S.Z.He	2015		77150616-1
	*Aspidistra yizhouensis* B.Pan & C.R.Lin	2016	IB	77152931-1
	*Aspidistra yunwuensis* S.Z.He & W.F.Xu	2015		77146882-1
	*Aspidistra zhenganensis* S.Z.He & Y.Wang	2017		77160874-1
	*Disporopsis bakerorum* Floden	2015	SW	60475820-2
	*Disporopsis yui* Floden	2015	IB	60475821-2
	*Disporum sinovietnamicum* R.C.Hu & Y.Feng Huang	2016	IB	77155596-1
	*Disporum xilingense* X.X.Zhu & Lin Zhang	2016	SW	77153735-1
	*Heteropolygonatum hainanense* Floden	2018	IB	77190162-1
	*Heteropolygonatum wugongshanense* G.X.Chen, Ying Meng & J.W.Xiao	2017		77177721-1
	*Ophiopogon yangshuoensis* R.H.Jiang & W.B.Xu	2013	IB	77134259-1
	*Peliosanthes minutiflora* N.Tanaka, J.Murata & S.K.Wu	2013	IB	77131481-1
	*Polygonatum campanulatum* G.W.Hu	2015	IB	77151629-1
	*Polygonatum dolichocarpum* M.N.Tamura, Fuse & Y.P.Yang	2014	SW	77144425-1
	*Polygonatum gongshanense* L.H.Zhao & X.J.He	2014	SW	77143846-1
	*Polygonatum luteoverrucosum* Floden	2015	H	77151663-1
	*Polygonatum sinopubescens* M.T.An, Yun Lin & Jia G.Wang	2016		77178692-1
	*Polygonatum undulatifolium* Floden	2018	H	77191727-1
	*Tricyrtis xianjuensis* G.Y.Li, Z.H.Chen & D.D.Ma	2014		77142359-1
	*Tupistra hongheensis* G.W.Hu & H.Li	2013	IB	77144640-1
Convolvulaceae	*Convolvulus xanthopotamicus* J.R.I.Wood & Scotland	2015		77147662-1
Corylaceae	*Carpinus insularis* N.H.Xia, K.S.Pang & Y.H.Tong	2014	IB	77140462-1
	*Carpinus langaoensis* Z.Qiang Lu & J.Quan Liu	2017		77160329-1
	*Carpinus tibetana* Z.Qiang Lu & J.Quan Liu	2018	SW	60476297-2
Crassulaceae	*Rhodiola daochengensis* J.Q.Zhang & G.Y.Rao	2015	SW	77149647-1
	*Rhodiola tricarpa* S.Y.Meng & G.Y.Rao	2015		77149655-1
	*Sedum kuntsunianum* X.F.Jin, S.H.Jin & B.Y.Ding	2013		77129875-1
	*Sedum spiralifolium* D.Q.Wang, D.M.Xie & Lu Q.Huang	2014		77143615-1
Cucurbitaceae	*Gomphogyne hainanensis* X.L.Zheng	2017	IB	77163861-1
	*Herpetospermum operculatum* K.Pradheep, A.Pandey, K.C.Bhatt & E.R.Nayar	2014	IB	77142405-1
	*Trichosanthes napoensis* D.X.Nong & Lu Q.Huang	2015	IB	77147130-1
Cycadaceae	*Cycas chenii* X.Gong & Wei Zhou	2015		77166521-1
Cyperaceae	*Carex bamaensis* X.F.Jin & W.Jie Chen	2015	IB	77150597-1
	*Carex concava* H.B.Yang, Xiao X.Li & G.D.Liu	2016	IB	77159299-1
	*Carex daxinensis* Y.Y.Zhou & X.F.Jin	2014	IB	77140321-1
	*Carex diaoluoshanica* H.B.Yang, G.D.Liu & Qing L.Wang	2014	IB	77137990-1
	*Carex fangiana* X.F.Jin & Y.Y.Zhou	2014	SW	77140322-1
	*Carex helingeeriensis* L.Q.Zhao & Jie Yang	2013		77130892-1
	*Carex honglinii* Y.F.Lu & X.F.Jin	2018		77191014-1
	*Carex huangshanica* X.F.Jin & W.J.Chen	2015		77150848-1
	*Carex jianfengensis* H.B.Yang, Xiao X.Li & G.D.Liu	2015	IB	77148926-1
	*Carex longicolla* Tang & F.T.Wang ex Y.F.Deng	2014	IB	77143317-1
	*Carex nodosa* S.R.Zhang, J.Zhang, Z.Y.Liu, S.Qu & R.G.Han	2018	SW	77192871-1
Cyperaceae	*Carex pararadicalis* X.F.Jin & J.M.Cen	2015	SW	77150598-1
	*Carex pengii* X.F.Jin & C.Z.Zheng	2013	IB	77132652-1
	*Carex procumbens* H.B.Yang, Xiao X.Li & G.D.Liu	2015	IB	77145896-1
	*Carex pseudomitrata* X.F.Jin & J.M.Cen	2015		77148010-1
	*Carex remotistachya* Y.Y.Zhou & X.F.Jin	2014		77140320-1
	*Carex scopulus* X.F.Jin & W.Jie Chen	2015		77150596-1
	*Carex sinosupina* Y.F.Lu & X.F.Jin	2017	SW	77174714-1
	*Carex tingnungii* X.F.Jin	2015		77150599-1
	*Carex xueyingiana* H.J.Yang & Han Xu	2017	IB	77163575-1
	*Fimbristylis longquanensis* X.F.Jin, Y.F.Lu & C.Z.Zheng	2017		77174068-1
	*Fimbristylis minuticulmis* X.F.Jin & C.Z.Zheng	2017		77174069-1
	*Sumatroscirpus rupestris* Lév.-Bourret & J.R.Starr	2018	IB	77194736-1
Dryopteridaceae	*Ctenitis jinfoshanensis* Ching & Z.Y.Liu	2015	SW	77146883-1
	*Dryopteris damingshanensis* Li Bing Zhang & Hong M.Liu	2014	IB	77141619-1
	*Dryopteris erythrovaria* K.Hori & N.Murak.	2018		77190681-1
	*Dryopteris shiakeana* H.Shang & Y.H.Yan	2015	IB	77148225-1
	*Dryopteris subtsushimensis* K.Hori & N.Murak.	2018		77194708-1
	*Polystichum alluvium* Li Bing Zhang, N.T.Lu & Yi F.Duan	2017		77177419-1
	*Polystichum deltatum* Li Bing Zhang, M.Q.Han & Yan Liu	2018	IB	77188039-1
	*Polystichum duyunense* Li Bing Zhang, X.Y.Miao & Chun X.Li	2017		77160176-1
	*Polystichum gejiuense* Li Bing Zhang, M.Q.Han & Yan Liu	2018	IB	77188040-1
	*Polystichum hainanicola* Li Bing Zhang, Liang Zhang & X.F.Gao	2013	IB	77125862-1
	*Polystichum hanmengqii* Li Bing Zhang & Yan Liu	2018	IB	77188041-1
	*Polystichum hastipinnum* G.D.Tang & Li Bing Zhang	2017	IB	77174044-1
	*Polystichum hubeiense* Liang Zhang & Li Bing Zhang	2013		77131021-1
	*Polystichum luteoviride* Li Bing Zhang, Yi F.Duan, N.T.Lu & Liang Zhang	2017		77176293-1
	*Polystichum malipoense* Li Bing Zhang, M.Q.Han & Yan Liu	2018	IB	77188042-1
	*Polystichum mulunense* X.L.Shen & R.H.Jiang	2015	IB	77153712-1
	*Polystichum muscicola* Ching ex W.M.Chu & Z.R.He	2013	SW	77133798-1
	*Polystichum oblongipinnarum* Li Bing Zhang, M.Q.Han & Yan Liu	2018	IB	77188043-1
	*Polystichum pingbianense* Li Bing Zhang, M.Q.Han & Yan Liu	2018	IB	77188044-1
	*Polystichum recavum* H.J.Wei & Li Bing Zhang	2018	IB	77191641-1
	*Polystichum rectum* Li Bing Zhang, M.Q.Han & Yan Liu	2018	IB	77188045-1
	*Polystichum superum* Li Bing Zhang, M.Q.Han & Yan Liu	2018	IB	77188046-1
	*Polystichum tiandengense* H.He & Li Bing Zhang	2017	IB	77175793-1
	*Polystichum zhijinense* Li Bing Zhang, Yi F.Duan & Kropf	2017		77174931-1
	*Tectaria × hongkongensis* S.Y.Dong	2016	IB	77155799-1
Ebenaceae	*Diospyros leei* Yan Liu, Song Shi & Y.S.Huang	2015	IB	77174862-1
	*Diospyros minutisepala* Kottaim.	2018	IB	77195755-1
Elaeocarpaceae	*Sloanea longiaculeatae* Y.F.Xie & Z.X.Zhang	2018	IB	77177789-1
Ericaceae	*Agapetes xiana* Y.H.Tong	2016	H	77153889-1
	*Cheilotheca crocea* L.Wu & Yan Liu	2016	IB	77155124-1
	*Gaultheria ciliisepala* Airy Shaw ex P.W.Fritsch & Lu Lu	2015	SW	77145871-1
	*Gaultheria gonggashanensis* P.W.Fritsch & Lu Lu	2015	SW	77154770-1
	*Gaultheria marronina* P.W.Fritsch & Lu Lu	2016	SW	77158467-1
	*Gaultheria stenophylla* P.W.Fritsch & Lu Lu	2015	SW	77145878-1
	*Rhododendron baihuaense* Y.P.Ma	2013	SW	77130537-1
	*Rhododendron bailiense* Y.P.Ma, C.Q.Zhang & D.F.Chamb.	2015		77144884-1
	*Rhododendron leigongshanense* C.H.Yang, Z.G.Xie, Y.F.Yu & Z.R.Yang	2015		77178680-1
	*Rhododendron longipedicellatum* Lei Cai & Y.P.Ma	2016	IB	77159155-1
	*Rhododendron microcarpum* R.L.Liu & L.M.Gao	2018		77185869-1
	*Rhododendron xiaoxueshanense* R.L.Liao & Y.P.Ma	2015	SW	77151708-1
	*Vaccinium damingshanense* Y.H.Tong & N.H.Xia	2014	IB	77146547-1
Euphorbiaceae	*Bischofia racemosa* W.C.Cheng & C.D.Chu ex Yi F.Duan & X.R.Wang	2016		77158535-1
	*Euphorbia maoershanensis* F.N.Wei & J.S.Ma	2013	IB	77127273-1
Euphorbiaceae	*Tsaiodendron dioicum* Y.H.Tan, Z.Zhou & B.J.Gu	2017	IB	77164524-1
Fagaceae	*Quercus pseudosetulosa* Q.S.Li & T.Y.Tu	2018	IB	77191553-1
Flacourtiaceae	*Flacourtia turbinata* H.J.Dong & H.Peng	2013	IB	77128867-1
Gentianaceae	*Swertia subuniflora* B.Hua Chen & Shi L.Chen	2016	IB	77159053-1
	*Tripterospermum maculatum* Adr.Favre, Matuszak & Muellner	2013	SW	77130745-1
Gesneriaceae	*Anna rubidiflora* S.Z.He, F.Wen & Y.G.Wei	2013		77130241-1
	*Briggsia leiophylla* F.Wen & Y.G.Wei	2015		77145932-1
	*Didissandra chishuiense* R.B.Zhang	2015		77153579-1
	*Didymocarpus anningensis* Y.M.Shui, Lei Cai & J.Cai	2015		77154513-1
	*Didymocarpus dissectus* F.Wen, Y.L.Qiu, Jie Huang & Y.G.Wei	2013	IB	77130005-1
	*Didymocarpus tonghaiensis* J.M.Li & F.S.Wang	2014		77146545-1
	*Hemiboea crystallina* Y.M.Shui & W.H.Chen	2018	IB	77176620-1
	*Hemiboea lutea* F.Wen, G.Y.Liang & Y.G.Wei	2013	IB	77135583-1
	*Hemiboea malipoensis* Y.H.Tan	2014	IB	77142385-1
	*Hemiboea roseoalba* S.B.Zhou, Xin Hong & F.Wen	2013	IB	77178534-1
	*Hemiboea suiyangensis* Z.Y.Li, S.W.Li & X.G.Xiang	2018		60476482-2
	*Loxostigma hekouense* Lei Cai, Gui L.Zhang & Z.L.Dao	2017	IB	77175302-1
	*Oreocharis brachypodus* J.M.Li & Zhi M.Li	2015		77146847-1
	*Oreocharis crispata* W.H.Chen & Y.M.Shui	2017	IB	77164334-1
	*Oreocharis curvituba* J.J.Wei & W.B.Xu	2016	IB	77159066-1
	*Oreocharis duyunensis* Z.Y.Li, X.G.Xiang & Z.Y.Guo	2018		77192883-1
	*Oreocharis jinpingensis* W.H.Chen & Y.M.Shui	2013	IB	77134255-1
	*Oreocharis ninglangensis* W.H.Chen & Y.M.Shui	2016	SW	77155208-1
	*Oreocharis ovata* L.H.Yang, L.X.Zhou & M.Kang	2018	IB	77193002-1
	*Oreocharis parviflora* Lei Cai & Z.K.Wu	2017	SW	77177745-1
	*Oreocharis purpurata* B.Pan, M.Q.Han & Yan Liu	2017		77177720-1
	*Oreocharis striata* F.Wen & C.Z.Yang	2015	IB	77174895-1
	*Oreocharis synergia* W.H.Chen, Y.M.Shui & Mich.Möller	2015	SW	77150839-1
	*Oreocharis tsaii* Y.H.Tan & Jian W.Li	2015	IB	77144882-1
	*Oreocharis uniflora* Li H.Yang & M.Kang	2017	IB	77160516-1
	*Oreocharis yunnanensis* Rossini & J.Freitas	2014	IB	77140287-1
	*Oreocharis zhenpingensis* J.M.Li, Ting Wang & Y.G.Zhang	2017		77163084-1
	*Paraboea crassifila* W.B.Xu & J.Guo	2016	IB	77154217-1
	*Paraboea dushanensis* W.B.Xu & M.Q.Han	2017		77174037-1
	*Paraboea sinovietnamica* W.B.Xu & J.Guo	2017	IB	77174038-1
	*Paraboea tetrabracteata* F.Wen, Xin Hong & Y.G.Wei	2013	IB	77133174-1
	*Paraboea wenshanensis* Xin Hong & F.Wen	2018	IB	60476045-2
	*Paraboea xiangguiensis* W.B.Xu & B.Pan	2017	IB	77174039-1
	*Paraboea yunfuensis* F.Wen & Y.G.Wei	2016	IB	77157473-1
	*Petrocodon ainsliifolius* W.H.Chen & Y.M.Shui	2014	IB	77142559-1
	*Petrocodon asterocalyx* F.Wen, Y.G.Wei & R.L.Zhang	2018	IB	77177603-1
	*Petrocodon confertiflorus* Hui Qin Li & Y.Q.Wang	2015	IB	77147097-1
	*Petrocodon hunanensis* X.L.Yu & Ming Li	2015		77144886-1
	*Petrocodon laxicymosus* W.B.Xu & Yan Liu	2014	IB	77141625-1
	*Petrocodon lithophilus* Y.M.Shui, W.H.Chen & Mich.Möller	2014		77142561-1
	*Petrocodon longgangensis* W.H.Wu & W.B.Xu	2014	IB	77141626-1
	*Petrocodon pseudocoriaceifolius* Yan Liu & W.B.Xu	2014	IB	77141627-1
	*Petrocodon pulchriflorus* Y.B.Lu & Q.Zhang	2017	IB	77160033-1
	*Petrocodon retroﬂexus* Qiang Zhang & J.Guo	2016		77155598-1
	*Petrocodon urceolatus* F.Wen, H.F.Cen & L.F.Fu	2017		77174676-1
	*Petrocodon villosus* Xin Hong, F.Wen & S.B.Zhou	2014	IB	77142626-1
	*Petrocodon viridescens* W.H.Chen, Mich.Möller & Y.M.Shui	2014	IB	77142562-1
	*Petrocosmea chrysotricha* M.Q.Han, H.Jiang & Yan Liu	2018	IB	77179090-1
	*Petrocosmea funingensis* Qiang Zhang & B.Pan	2013	IB	77125525-1
	*Petrocosmea glabristoma* Z.J.Qiu & Yin Z.Wang	2015	IB	77151734-1
	*Petrocosmea magnifica* M.Q.Han & Yan Liu	2017		77176611-1
Gesneriaceae	*Petrocosmea melanophthalma* Huan C.Wang, Z.R.He & Li Bing Zhang	2013	IB	77137989-1
	*Petrocosmea viridis* M.Q.Han & Yan Liu	2017		77179033-1
	*Primulina albicalyx* B.Pan & Li H.Yang	2017	IB	77175151-1
	*Primulina alutacea* F.Wen, B.Pan & B.M.Wang	2016	IB	77153658-1
	*Primulina argentea* Xin Hong, F.Wen & S.B.Zhou	2014	IB	77144595-1
	*Primulina beiliuensis* B.Pan & S.X.Huang	2013	IB	77135659-1
	*Primulina bullata* S.N.Lu & F.Wen	2013	IB	77128701-1
	*Primulina cangwuensis* Xin Hong & F.Wen	2018	IB	77186304-1
	*Primulina carinata* Y.G.Wei, F.Wen & H.Z.Lü	2014	IB	77147651-1
	*Primulina cordistigma* F.Wen, B.D.Lai & B.M.Wang	2016	IB	77152079-1
	*Primulina crassirhizoma* F.Wen, Bo Zhao & Xin Hong	2013	IB	77178533-1
	*Primulina curvituba* B.Pan, L.H.Yang & M.Kang	2017	IB	77174715-1
	*Primulina davidioides* F.Wen & Xin Hong	2018	IB	77179004-1
	*Primulina debaoensis* N.Jiang & Hong Li	2013	IB	77133646-1
	*Primulina dichroantha* F.Wen, Y.G.Wei & S.B.Zhou	2017	IB	77163880-1
	*Primulina diffusa* Xin Hong, F.Wen & S.B.Zhou	2014	IB	77142358-1
	*Primulina dongguanica* F.Wen, Y.G.Wei & R.Q.Luo	2014	IB	77141126-1
	*Primulina duanensis* F.Wen & S.L.Huang	2014	IB	77148862-1
	*Primulina effusa* F.Wen & B.Pan	2017	IB	77175213-1
	*Primulina fengkaiensis* Z.L.Ning & M.Kang	2015	IB	77146194-1
	*Primulina gigantea* F.Wen, B.Pan & W.H.Luo	2016	IB	77159842-1
	*Primulina glandaceistriata* X.X.Zhu, F.Wen & H.Sun	2014	IB	77143943-1
	*Primulina guizhongensis* Bo Zhao, B.Pan & F.Wen	2013	IB	60461605-2
	*Primulina hengshanensis* L.H.Liu & K.M.Liu	2018		77176509-1
	*Primulina heterochroa* F.Wen & B.D.Lai	2015	IB	77147015-1
	*Primulina hiemalis* Xin Hong & F.Wen	2018	IB	77179005-1
	*Primulina huaijiensis* Z.L.Ning & Jing Wang	2013	IB	77131011-1
	*Primulina huangii* F.Wen & Z.B.Xin	2018	IB	77178940-1
	*Primulina hunanensis* K.M.Liu & X.Z.Cai	2015		77154763-1
	*Primulina jianghuaensis* K.M.Liu & X.Z.Cai	2013		77137130-1
	*Primulina jiangyongensis* X.L.Yu & Ming Li	2014		77143055-1
	*Primulina lechangensis* Xin Hong, F.Wen & S.B.Zhou	2014	IB	77178668-1
	*Primulina lepingensis* Z.L.Ning & Ming Kang	2014		77143841-1
	*Primulina linearicalyx* F.Wen, B.D.Lai & Y.G.Wei	2016	IB	77156700-1
	*Primulina lutescens* B.Pan & H.S.Ma	2017	IB	77174730-1
	*Primulina lutvittata* F.Wen & Y.G.Wei	2013	IB	77130904-1
	*Primulina mabaensis* K.F.Chung & W.B.Xu	2013	IB	77127679-1
	*Primulina maciejewskii* F.Wen, R.L.Zhang & A.Q.Dong	2016	IB	77161526-1
	*Primulina maculata* W.B.Xu & J.Guo	2015	IB	77156518-1
	*Primulina malipoensis* Li H.Yang & M.Kang	2018	IB	77175494-1
	*Primulina melanofilamenta* Y.Liu & F.Wen	2016	IB	77154836-1
	*Primulina minor* F.Wen & Y.G.Wei	2013		77143404-1
	*Primulina moi* F.Wen & Y.G.Wei	2015	IB	77150285-1
	*Primulina pengii* W.B.Xu & K.F.Chung	2015	IB	77156519-1
	*Primulina petrocosmeoides* B.Pan & F.Wen	2014	IB	77144566-1
	*Primulina porphyrea* X.L.Yu & Ming Li	2015		77147207-1
	*Primulina pseudoroseoalba* Jian Li, F.Wen & L.J.Yan	2014	IB	77139170-1
	*Primulina qingyuanensis* Z.L.Ning & Ming Kang	2013	IB	77133454-1
	*Primulina rubella* L.H.Yang & M.Kang	2017	IB	77174703-1
	*Primulina rubribracteata* Z.L.Ning & M.Kang	2015		77151713-1
	*Primulina suichuanensis* X.L.Yu & J.J.Zhou	2016		77155595-1
	*Primulina tsoongii* H.L.Liang, Bo Zhao & F.Wen	2013	IB	77137131-1
	*Primulina versicolor* F.Wen, B.Pan & B.M.Wang	2016	IB	77153657-1
	*Primulina wenii* Jian Li & L.J.Yan	2017	IB	77178746-1
	*Primulina wuae* F.Wen & L.F.Fu	2017	IB	77175898-1
	*Primulina yandongensis* Ying Qin & Yan Liu	2018	IB	77193180-1
Gesneriaceae	*Primulina yangchunensis* Y.L.Zheng & Y.F.Deng	2014	IB	77140273-1
	*Primulina yangshanensis* W.B.Xu & B.Pan	2015	IB	77156520-1
	*Primulina yingdeensis* Z.L.Ning, M.Kang & X.Y.Zhuang	2016	IB	77159797-1
	*Primulina zhoui* F.Wen & Z.B.Xin	2018	IB	77178939-1
	*Raphiocarpus jinpingensis* W.H.Chen & Y.M.Shui	2015	IB	77153711-1
	*Tremacron hongheense* W.H.Chen & Y.M.Shui	2015	IB	77151819-1
Grammitidaceae	*Oreogrammitis hainanensis* Parris	2013	IB	77133850-1
	*Oreogrammitis orientalis* T.C.Hsu	2017	IB	77162889-1
	*Oreogrammitis sinohirtella* Parris	2013	IB	77133851-1
Hydrocharitaceae	*Ottelia guanyangensis* Z.Z.Li, Q.F.Wang & S.Wu	2018	IB	77187252-1
Lamiaceae	*Isodon aurantiacus* Y.P.Chen & C.L.Xiang	2017	H	77175276-1
	*Isodon delavayi* C.L.Xiang & Y.P.Chen	2014	SW	77137826-1
	*Isodon villosus* Y.P.Chen & H.Peng	2016	IB	77156696-1
	*Meehania hongliniana* B.Y.Ding & X.F.Jin	2018		77193006-1
	*Nepeta wuana* H.J.Dong, C.L.Xiang & Jamzad	2015		77150066-1
	*Pogostemon henanensis* Gang Yao	2015		77145867-1
	*Salvia lagochila* T.Wang & L.Wang	2016	SW	77154837-1
	*Salvia luteistriata* G.X.Hu, E.D.Liu & C.L.Xiang	2017	SW	77176301-1
	*Salvia petrophila* G.X.Hu, E.D.Liu & Yan Liu	2013	IB	77138690-1
	*Scutellaria wuana* C.L.Xiang & F.Zhao	2017	SW	77164113-1
Lauraceae	*Alseodaphnopsis ximengensis* H.W.Li & J.Li	2017	IB	77165686-1
	*Beilschmiedia turbinata* Bing Liu & Y.Yang	2013	IB	77128395-1
	*Caryodaphnopsis malipoensis* Bing Liu & Y.Yang	2013	IB	77130681-1
	*Litsea dorsalicana* M.Q.Han & Y.S.Huang	2013	IB	77130684-1
Leguminosae	*Astragalus animaqingshanicus* Y.H.Wu	2015		77157961-1
	*Astragalus gandeensis* Y.H.Wu	2015		77157958-1
	*Astragalus jigenensis* Y.H.Wu	2015	CA	77157967-1
	*Astragalus magnibracteus* Y.H.Wu	2015	CA	77157955-1
	*Astragalus majixueshanicus* Y.H.Wu	2015		77157959-1
	*Astragalus maquensis* Y.H.Wu	2015	SW	77157963-1
	*Astragalus nuoergongensis* L.Q.Zhao & Xu Ri	2018		77193175-1
	*Astragalus wulingensis* Jia X.Li & X.L.Yu	2014		77138130-1
	*Astragalus xijiangensis* L.R.Xu & Y.H.Wu	2015	CA	77157965-1
	*Bauhinia hekouensis* T.Y.Tu & D.X.Zhang	2013	IB	77132598-1
	*Dalbergia changhuagensis* G.A.Fu, Y.K.Yang & Wen Q.Wang	2015	IB	77165106-1
	*Hedysarum cuonanum* P.L.Liu, J.Wen & Zhao Y.Chang	2017	H	77160511-1
	*Hedysarum xichuanicum* Y.H.Wu	2015	SW	77157997-1
	*Hedysarum yushuensis* Y.H.Wu	2015		77157996-1
	*Indigofera pseudonigrescens* X.F.Gao & X.L.Zhao	2015	SW	77149467-1
	*Lespedeza jiangxiensis* Bo Xu, X.F.Gao & Li Bing Zhang	2013		77130627-1
	*Lespedeza pseudomaximowiczii* D.P.Jin, Bo Xu & B.H.Choi	2018		77193152-1
	*Oxytropis anyemaqensis* Y.H.Wu	2015		77157976-1
	*Oxytropis barunensis* Y.H.Wu	2015		77157980-1
	*Oxytropis burhanbudaica* Y.H.Wu	2015		77157974-1
	*Oxytropis datongensis* Y.H.Wu	2015		77157995-1
	*Oxytropis gandeensis* Y.H.Wu	2015		77157975-1
	*Oxytropis huashixiaensis* Y.H.Wu	2015		77157978-1
	*Oxytropis qaidamensis* Y.H.Wu	2015		77157970-1
	*Oxytropis xidatanensis* Y.H.Wu	2015		77157977-1
	*Oxytropis xinghaiensis* Y.H.Wu	2015		77157972-1
	*Oxytropis zadoiensis* Y.H.Wu	2015		77157979-1
	*Oxytropis zaquensis* Y.H.Wu	2015		77157993-1
	*Pseudarthria panii* R.Zhang, T.S.Yi & B.Pan bis	2018	IB	77190130-1
	*Pueraria grandiflora* B.Pan bis & Bing Liu	2015	SW	77145972-1
	*Vicia aktoensis* Y.H.Wu	2015	CA	77157999-1
Liliaceae	*Amana wanzhensis* Lu Q.Huang, B.X.Han & K.Zhang	2014		77143001-1
Liliaceae	*Lilium yapingense* Y.D.Gao & X.J.He	2013	SW	77130239-1
	*Saussurea bogedaensis* Yu J.Wang & J.Chen	2018	CA	77180814-1
Lowiaceae	*Orchidantha yunnanensis* P.Zou, C.F.Xiao & Škorničk.	2017	IB	77162277-1
Lycopodiaceae	*Huperzia nanlingensis* Y.H.Yan & N.Shrestha	2014	IB	77141799-1
	*Huperzia shresthae* Fraser-Jenk.	2018	IB	77191157-1
	*Lycopodium verticale* Li Bing Zhang	2013	SW	77133467-1
	*Spinulum lioui* Li Bing Zhang & H.He	2017		77163016-1
Lythraceae	*Lagerstroemia densa* C.H.Gu & D.D.Ma	2015	IB	77146188-1
Magnoliaceae	*Magnolia kwangnanensis* S.G.Chen & Q.W.Zeng	2013	IB	77131015-1
	*Magnolia tiepii* V.T.Tran & Duy	2015	IB	77150281-1
	*Manglietia admirabilis* Y.W.Law & R.Z.Zhou ex L.Fu, Q.W.Zeng & X.M.Hu	2014	IB	77141391-1
	*Manglietia guangnanica* D.X.Li & R.Z.Zhou ex X.M.Hu, Q.W.Zeng & L.Fu	2014	IB	77147528-1
Malpighiaceae	*Hiptage ferruginea* Y.H.Tan & Bin Yang	2018	IB	77191588-1
	*Hiptage pauciflora* Y.H.Tan & Bin Yang	2018	IB	77191587-1
Malvaceae	*Malva xizangensis* Y.S.Ye, L.Fu & D.X.Duan	2015	SW	77147329-1
Marantaceae	*Phrynium yunnanense* Y.S.Ye & L.Fu	2017	IB	77163010-1
Melanthiaceae	*Chamaelirium viridiflorum* Lei Wang, Z.C.Liu & W.B.Liao	2018		77185949-1
	*Chionographis nanlingensis* L.Wu, Y.Tong & Q.R.Liu	2016	IB	77155663-1
Melastomataceae	*Bredia changii* W.Y.Zhao, X.H.Zhan & W.B.Liao	2017		77163004-1
	*Bredia repens* R.Zhou, Q.J.Zhou & Ying Liu	2018		77194739-1
	*Fordiophyton chenii* S.Jin Zeng & X.Y.Zhuang	2016	IB	77153256-1
	*Fordiophyton huizhouense* S.Jin Zeng & X.Y.Zhuang	2016	IB	77153257-1
	*Fordiophyton zhuangiae* S.Jin Zeng & G.D.Tang	2016	IB	77159141-1
	*Phyllagathis guidongensis* K.M.Liu & J.Tian	2016		77161451-1
	*Sonerila trinervis* Q.W.Lin	2015	IB	77151737-1
Menispermaceae	*Stephania novenanthera* Heng C.Wang	2013	IB	77132616-1
Moraceae	*Ficus cornelisiana* Chantaras. & Y.Q.Peng	2014	IB	77142617-1
Musaceae	*Musa ruiliensis* W.N.Chen, Häkkinen & X.J.Ge	2014	IB	77141452-1
Myrsinaceae	*Ardisia bullata* G.H.Huang & G.Hao	2018	IB	77193172-1
	*Ardisia medogensis* C.M.Hu & G.Hao	2018	H	77191512-1
	*Ardisia nutantiflora* S.Z.Mao & C.M.Hu	2018	IB	77193157-1
	*Ardisia rubricaulis* S.Z.Mao & C.M.Hu	2013	IB	77133457-1
	*Sadiria longistyla* Ze H.Wang & H.Peng	2018	IB	77177770-1
Orchidaceae	*Anoectochilus dulongensis* Ormerod	2013	SW	77137327-1
	*Anoectochilus longilobus* H.Jiang & H.Z.Tian	2014	IB	77140335-1
	*Anoectochilus nandanensis* Y.Feng Huang & X.C.Qu	2015	IB	77154757-1
	*Apostasia fogangica* Y.Y.Yin, P.S.Zhong & Z.J.Liu	2016	IB	77158583-1
	*Arachnis bouffordii* Ormerod	2014	IB	77136159-1
	*Bulbophyllum chrysolabium* L.Li & D.P.Ye	2018	IB	77191936-1
	*Bulbophyllum huangshanense* Y.M.Hu & X.H.Jin	2015		77150284-1
	*Bulbophyllum jingdongense* A.Q.Hu, D.P.Ye & Jian W.Li	2017	IB	77163074-1
	*Bulbophyllum lipingtaoi* Jiu X.Huang, J.Y.Wang & Z.J.Liu	2017	IB	77160509-1
	*Bulbophyllum menglaense* Jian W.Li & X.H.Jin	2017	IB	77164286-1
	*Bulbophyllum mengyuanense* Q.Liu, Jian W.Li & X.H.Jin	2015	IB	77151668-1
	*Bulbophyllum nujiangense* X.H.Jin & W.T.Jin	2014	SW	77141124-1
	*Bulbophyllum pingnanense* J.F.Liu, S.R.Lan & Y.C.Liang	2016	IB	77155742-1
	*Bulbophyllum salweenensis* X.H.Jin	2015	SW	77150855-1
	*Bulbophyllum yingjiangense* B.M.Wang & J.W.Zhai	2017	IB	77161275-1
	*Bulbophyllum yongtaiense* J.F.Liu, S.R.Lan & Y.C.Liang	2018	IB	77178918-1
	*Bulbophyllum yunxiaoense* M.H.Li, J.F.Liu & S.P.Chen	2017	IB	77174896-1
	*Calanthe bingtaoi* J.W.Zhai, L.J.Chen & Z.J.Liu	2013	SW	77132014-1
	*Calanthe longgangensis* Y.S.Huang & Yan Liu	2015	IB	77149688-1
	*Calanthe taibaishanensis* M.Guo, J.W.Zhai & L.J.Chen	2017		77177695-1
	*Calanthe wenshanensis* J.W.Zhai, L.J.Chen & Z.J.Liu	2013	IB	77132013-1
Orchidaceae	*Calanthe wuxiensis* H.P.Deng & F.Q.Yu	2017	SW	77176471-1
	*Cestichis pingtaoi* G.D.Tang, X.Y.Zhuang & Z.J.Liu	2015	IB	77151433-1
	*Changnienia malipoensis* D.H.Peng, Z.J.Liu & J.W.Zhai	2013	IB	77130095-1
	*Cheirostylis acuminata* Zhi L.Liu & Q.Liu	2016	IB	77158509-1
	*Coelogyne pianmaensis* R.Li & Z.L.Dao	2014	SW	77138678-1
	*Collabium yunnanense* Ormerod	2013	SW	77137331-1
	*Cremastra malipoensis* G.W.Hu	2013	IB	77130401-1
	*Cymbidium daweishanense* G.Q.Zhang & Z.J.Liu	2018	IB	77191723-1
	*Cymbidium lii* M.Z.Huang, J.M.Yin & G.S.Yang	2017	IB	77176352-1
	*Cymbidium puerense* Z.J.Liu & S.R.Lan	2018	IB	77185942-1
	*Danxiaorchis singchiana* J.W.Zhai, F.W.Xing & Z.J.Liu	2013	IB	77124909-1
	*Danxiaorchis yangii* Bo Y.Yang & Bo Li	2017		77162990-1
	*Dendrobium bannaense* Y.Q.Tian & Y.B.Huang	2017	IB	77177715-1
	*Dendrobium libingtaoi* Q.Xu & Z.J.Liu	2018	IB	77176514-1
	*Dendrobium longlingense* Q.Xu, Y.B.Luo & Z.J.Liu	2014	IB	77142382-1
	*Dendrobium luoi* L.J.Chen & W.H.Rao	2016		77155442-1
	*Dendrobium maguanense* Q.Xu & Z.J.Liu	2016	IB	77159344-1
	*Dendrobium wenshanense* Q.Xu, Y.B.Luo & Z.J.Liu	2014	IB	77142381-1
	*Dendrobium zhenghuoense* S.P.Chen, Liang Ma & Ming H.Li	2016	IB	77158455-1
	*Dendrobium zhenyuanense* D.P.Ye ex Jian W.Li, D.P.Ye & X.H.Jin	2014	IB	77143319-1
	*Gastrochilus dulongjiangensis* Q.Liu & J.Y.Gao	2018	SW	77177530-1
	*Gastrochilus jietouensis* Ormerod	2013	SW	77137333-1
	*Gastrochilus kadooriei* Kumar, S.W.Gale, Kocyan, G.A.Fisch. & Aver.	2014	IB	77140315-1
	*Gastrochilus tianbaoensis* Q.Liu & Y.H.Tan	2016	IB	77159092-1
	*Gastrodia damingshanensis* A.Q.Hu & T.C.Hsu	2014	IB	77142938-1
	*Gastrodia huapingensis* X.Y.Huang, A.Q.Hu & Yan Liu	2015	IB	77149471-1
	*Goodyera makuensis* Ormerod	2013	SW	77137395-1
	*Goodyera malipoensis* Q.X.Guan & S.P.Chen	2014	IB	77143809-1
	*Habenaria fimbriatiloba* Kolan.	2015		77145880-1
	*Habenaria luquanensis* G.W.Hu	2015		77151687-1
	*Habenaria malipoensis* Q.Liu & W.L.Zhang	2017	IB	77174902-1
	*Habenaria pseudorostellifera* Kolan., Szlach. & Kras	2015		77174894-1
	*Habenaria yachangensis* Z.B.Zhang & W.Guo	2015	IB	77144532-1
	*Hemipilia galeata* Ying Tang, X.X.Zhu & H.Peng	2016	IB	77158484-1
	*Herminium gongganum* Ormerod	2013	SW	77137396-1
	*Herminium motuoense* X.H.Jin	2017	H	77177751-1
	*Herminium tibeticum* X.H.Jin, Schuit. & Raskoti	2017	H	77161963-1
	*Holcoglossum singchianum* G.Q.Zhang, L.J.Chen & Z.J.Liu	2013	IB	77125663-1
	*Holopogon pekinensis* X.Y.Mu & Bing Liu	2017		77177662-1
	*Hygrochilus tsii* M.H.Li, Z.J.Liu & S.R.Lan	2014		77138128-1
	*Liparis funingensis* Yong Y.Su, Yuan Meng & Z.J.Liu	2014	IB	77141198-1
	*Liparis meihuashanensis* S.M.Fan	2017	IB	77177439-1
	*Liparis pingxiangensis* L.Li & H.F.Yan	2013	IB	77131431-1
	*Liparis tsii* H.Z.Tian & A.Q.Hu	2015	IB	77159236-1
	*Liparis vivipara* H.X.Huang, Z.J.Liu & M.H.Li	2018	IB	77180815-1
	*Liparis wenshanensis* Yong Y.Su, Yi L.Huang & G.Q.Zhang	2015	IB	77146844-1
	*Malaxis malipoensis* Y.F.Meng, A.Q.Hu & F.W.Xing	2014	IB	77140780-1
	*Malleola tibetica* W.C.Huang & X.H.Jin	2013	H	77135582-1
	*Neottia bicallosa* X.H.Jin	2014	SW	77143043-1
	*Neottia nujiangensis* X.H.Jin	2016	SW	77159994-1
	*Nervilia brevilobata* C.S.Leou, C.L.Yeh & S.W.Gale	2013		77131195-1
	*Odontochilus napoensis* H.Tang & Y.F.Huang	2016	IB	77157455-1
	*Oreorchis yachangensis* Z.B.Zhang & B.G.Huang	2016	IB	77155582-1
	*Paphiopedilum notatisepalum* Z.J.Liu, Meina Wang & S.R.Lan	2017	IB	77162273-1
	*Pendulorchis gaoligongense* G.Q.Zhang, K.Wei Liu & Z.J.Liu	2013	SW	77125661-1
	*Phalaenopsis pingxiangensis* Hua Deng, Z.J.Liu & Yan Wang	2015	IB	77151701-1
Orchidaceae	*Platanthera anatina* Ormerod	2013	SW	77137397-1
	*Platanthera australis* L.Wu, X.L.Yu, H.Z.Tian & J.L.Luo	2017		77163273-1
	*Platanthera danghatuensis* Ormerod	2013	SW	77137401-1
	*Platanthera fugongensis* Ormerod	2013	SW	77137412-1
	*Platanthera fujianensis* B.H.Chen & X.H.Jin	2016	IB	77159362-1
	*Platanthera guangdongensis* Y.F.Li, L.F.Wu & L.J.Chen	2018	IB	77177592-1
	*Platanthera nanlingensis* X.H.Jin & W.T.Jin	2015	IB	77174831-1
	*Platanthera yadongensis* X.H.Jin & W.T.Jin	2014	H	77136231-1
	*Platanthera zijinensis* Q.L.Ye, Z.M.Zhong & Ming H.Li	2018	IB	77177593-1
	*Platystyliparis malipoensis* G.D.Tang, X.Y.Zhuang & Z.J.Liu	2015	IB	77151432-1
	*Pleione × baoshanensis* W.Zhang & S.B.Zhang	2018	SW	77179038-1
	*Pleione × maoershanensis* W.Zhang & S.B.Zhang	2018	IB	77179039-1
	*Pleione jinhuana* Z.J.Liu, M.T.Jiang & S.R.Lan	2018		77177709-1
	*Spiranthes himalayensis* Survesw., Kumar & Mei Sun	2017	IB	77167300-1
	*Thuniopsis cleistogama* L.Li, D.P.Ye & Shi J.Li	2015	IB	77147753-1
	*Vanda funingensis* L.H.Zou & Z.J.Liu	2016	IB	77154813-1
	*Vanda malipoensis* L.H.Zou, Jiu X.Huang & Z.J.Liu	2014	IB	77143831-1
	*Yunorchis pingbianensis* Z.J.Liu, G.Q.Zhang & Ming H.Li	2015	IB	77145332-1
	*Zeuxine ovalifolia* L.Li & S.J.Li	2013	IB	77132060-1
Oxalidaceae	*Oxalis wulingensis* T.Deng, D.G.Zhang & Z.L.Nie	2013		77130727-1
Papaveraceae	*Corydalis hegangensis* W.T.Wang	2017		77175066-1
	*Corydalis hualongshanensis* D.Wang	2017		77163013-1
	*Corydalis huangshanensis* L.Q.Huang & H.S.Peng	2018		77192894-1
	*Corydalis latilepidota* W.T.Wang	2017	SW	77175065-1
	*Corydalis pseudoamplisepala* D.Wang	2017		77175263-1
	*Corydalis pseudohemsleyana* D.Wang	2017		77174716-1
	*Meconopsis × kongboensis* Grey-Wilson	2014		77143504-1
	*Meconopsis lhasaensis* Grey-Wilson	2014		77143508-1
	*Meconopsis zhongdianensis* Grey-Wilson	2014	SW	77143509-1
Phytolaccaceae	*Phytolacca exiensis* D.G.Zhang, L.Q.Huang & D.Xie	2017		77174910-1
Pinaceae	*Picea neohirtella* Silba	2015	SW	77150057-1
Piperaceae	*Piper jianfenglingense* C.Y.Hao & Y.H.Tan	2017	IB	77174928-1
	*Piper magen* B.Q.Cheng ex C.L.Long & Jun Yang bis	2017	IB	77176453-1
	*Piper peltatifolium* C.Y.Hao, H.S.Wu & Y.H.Tan	2015	IB	77151669-1
Poaceae	*Achnatherum pilosum* Z.S.Zhang & W.L.Chen	2018	SW	77178922-1
	*Capillipedium alpinum* H.Sun & Boufford	2016	SW	60477087-2
	*Dendrocalamus atroviridis* D.Z.Li & H.Q.Yang	2016	IB	77151941-1
	*Dendrocalamus jinghongensis* P.Y.Wang, Y.X.Zhang & D.Z.Li	2016	IB	77157469-1
	*Dendrocalamus longiauritus* S.H.Chen, K.F.Huang & R.S.Chen	2013	IB	77138281-1
	*Dendrocalamus yingjiangensis* D.Z.Li & H.Q.Yang	2015	IB	77174758-1
	*Deyeuxia gaoligongensis* Paszko	2013	SW	77127682-1
	*Deyeuxia sorengii* Paszko & W.L.Chen	2013		77133459-1
	*Elymus dolichorhachis* S.L.Lu & Y.H.Wu	2013		77135533-1
	*Elymus qingnanensis* S.L.Lu & Y.H.Wu	2013		77135534-1
	*Elymus zadoiensis* S.L.Lu & Y.H.Wu	2013		77135532-1
	*Fargesia microauriculata* M.S.Sun, D.Z.Li & H.Q.Yang	2016	SW	77159818-1
	*Fargesia weiningensis* T.P.Yi & Lin Yang	2013		77132561-1
	*Gelidocalamus xunwuensis* W.G.Zhang & G.Y.Yang	2017		77165358-1
	*Gigantochloa callosa* N.H.Xia, Y.Zeng & R.S.Lin	2014	IB	77144152-1
	*Holttumochloa hainanensis* M.Y.Zhou & D.Z.Li	2017	IB	77174625-1
	*Leymus golmudensis* Y.H.Wu	2013		77135531-1
	*Oligostachyum heterophyllum* M.M.Lin	2017	IB	77176053-1
	*Orinus intermedius* X.Su & J.Quan Liu	2017	SW	77175809-1
	*Phyllostachys acutiligula* G.H.Lai	2013		77131122-1
	*Phyllostachys corrugata* G.H.Lai	2013		77131124-1
	*Phyllostachys hirtivagina* G.H.Lai	2013		77131123-1
Poaceae	*Phyllostachys purpureociliata* G.H.Lai	2013		77131125-1
	*Pseudosasa xishuangbannaensis* D.Z.Li, Y.X.Zhang & Triplett	2013	IB	77134702-1
	*Ptilagrostis arcuata* Z.S.Zhang & W.L.Chen	2016	SW	77157281-1
	*Ptilagrostis contracta* Z.S.Zhang & W.L.Chen	2017	SW	77158315-1
	*Schizostachyum longinternodium* N.H.Xia, R.S.Lin & C.H.Zheng	2014	IB	77143644-1
	*Stipa dickorei* M.Nobis	2016		77155874-1
	*Stipa zhadaensis* L.Q.Zhao & K.Guo	2017		77163865-1
	*Yushania gigantea* T.P.Yi & L.Yang	2014	SW	77135675-1
	*Yushania pianmaensis* T.P.Yi & L.Yang	2014	SW	77135676-1
Podocarpaceae	*Podocarpus hookeri* de Laub.	2015	IB	77153935-1
	*Cladopus yinggelingensis* Q.W.Lin, Gang Lu & Z.Y.Li	2016	IB	77157336-1
	*Terniopsis daoyinensis* Q.W.Lin, Gang Lu & Z.Y.Li	2016	IB	77157337-1
Polygonaceae	*Fagopyrum hailuogouense* J.R.Shao, M.L.Zhou & Qian Zhang	2015	SW	60471656-2
	*Fagopyrum longzhoushanense* J.R.Shao	2017	SW	77160031-1
	*Fagopyrum luojishanense* J.R.Shao	2015	SW	77153445-1
	*Koenigia chuanzangensis* Z.Z.Zhou & Y.J.Min	2015	SW	77155422-1
	*Koenigia hedbergii* Bo Li & W.Du	2016		77157437-1
	*Persicaria changhuaensis* H.W.Zhang & X.F.Jin	2017		77174677-1
	*Persicaria lankeshanensis* T.J.Liang & Bo Li	2014	IB	77178666-1
	*Persicaria wugongshanensis* Bo Li	2014		77136296-1
Polypodiaceae	*Lepisorus simulans* Ching & Z.Y.Liu	2015	SW	77146884-1
	*Leptochilus mengsongensis* M.X.Zhao	2017	IB	77176470-1
Potamogetonaceae	*Ruppia brevipedunculata* Shuo Yu & Hartog	2014		77142902-1
	*Ruppia sinensis* Shuo Yu & Hartog	2014		77142903-1
Primulaceae	*Lysimachia dabieshanensis* Kun Liu & S.B.Zhou	2014		77142378-1
	*Lysimachia huangsangensis* J.J.Zhou, X.L.Yu & Y.F.Deng	2015		77147698-1
	*Lysimachia jinzhaiensis* S.B.Zhou & Kun Liu	2014		77141573-1
	*Lysimachia septemfida* Ze H.Wang & E.D.Liu	2016	IB	77165309-1
	*Lysimachia sinopilosa* C.M.Hu & G.Hao	2017	IB	77163864-1
	*Lysimachia tianmaensis* Kun Liu, S.B.Zhou & Ying Wang	2018		77178766-1
	*Primula anthemifolia* G.Hao, C.M.Hu & Yuan Xu	2015	SW	77174897-1
	*Primula centellifolia* G.Hao, C.M.Hu & Y.Xu	2017	SW	77177673-1
	*Primula chimingiana* G.Hao, S.Yuan & D.X.Zhang	2017	SW	77179003-1
	*Primula dejuniana* G.Hao, C.M.Hu & Yuan Xu	2014	SW	77145096-1
	*Primula hubeiensis* Xin W.Li	2017		77175796-1
	*Primula hunanensis* G.Hao, C.M.Hu & X.L.Yu	2014		77148873-1
	*Primula hydrocotylifolia* G.Hao, C.M.Hu & Yuan Xu	2015	SW	77148008-1
	*Primula jiugongshanensis* J.W.Shao	2017		77174675-1
	*Primula luteoflora* X.F.Gao & W.B.Ju	2018	SW	77190134-1
	*Primula mianyangensis* G.Hao & C.M.Hu	2013	SW	77133180-1
	*Primula pelargoniifolia* G.Hao, C.M.Hu & Z.Y.Liu	2014	SW	77139176-1
	*Primula pengzhouensis* C.M.Hu, G.Hao & Y.Xu	2017	SW	77174626-1
	*Primula persimilis* G.Hao, C.M.Hu & Y.Xu	2016	SW	77157456-1
	*Primula scopulicola* G.Hao, C.M.Hu & Y.Xu	2016	SW	77159817-1
	*Primula undulifolia* G.Hao, C.M.Hu & Y.Xu	2016		60472693-2
	*Primula wawushanica* G.Hao, C.M.Hu & Yuan Xu	2016	SW	77155597-1
	*Primula zhui* Y.H.Tan & B.Yang	2017	IB	77174729-1
	*Stimpsonia nanlingensis* G.H.Huang & G.Hao	2017	IB	77174635-1
Proteaceae	*Helicia yangchunensis* H.S.Kiu	2013	IB	77132615-1
Pteridaceae	*Pteris dixitii* Fraser-Jenk. & Pariyar	2015		77161778-1
Ranunculaceae	*Aconitum basitruncatum* W.T.Wang	2014	SW	77141210-1
	*Aconitum hezuoense* W.T.Wang	2015	SW	77149689-1
	*Aconitum lianhuashanicum* W.T.Wang	2015	SW	77149690-1
	*Aconitum luanchuanense* W.T.Wang	2015		77149687-1
	*Aconitum novoaxillare* W.T.Wang	2014	SW	77141209-1
	*Aconitum qianxiense* W.T.Wang	2013		77134723-1
Ranunculaceae	*Aconitum rotundocassideum* W.T.Wang	2013		77138280-1
	*Aconitum tuoliense* W.T.Wang	2016	CA	77160240-1
	*Aconitum wumengense* J.He & E.D.Liu	2018		77177553-1
	*Actaea muliensis* J.P.Luo, Q.E.Yang & Q.Yuan	2016	SW	77161534-1
	*Anemone brachystema* W.T.Wang	2014	H	77142643-1
	*Anemone jiachaensis* W.T.Wang	2014		77142644-1
	*Anemone milinensis* W.T.Wang	2013		77135654-1
	*Anemone motuoensis* W.T.Wang	2014	H	77142642-1
	*Aquilegia hebeica* Erst	2017		77176452-1
	*Aquilegia xinjiangensis* Erst	2017	CA	77176451-1
	*Aquilegia yangii* Y.Luo & Lu Li	2018	SW	77178664-1
	*Caltha dysosmoides* Tao Zhang, Bing Liu, Y.Q.Hao, Y.Yang & Y.J.Lai	2016	SW	77159219-1
	*Clematis chaohuensis* W.T.Wang & L.Q.Huang	2014		77140156-1
	*Clematis diebuensis* W.T.Wang	2015	SW	77145271-1
	*Clematis dongchuanensis* W.T.Wang	2014		77142362-1
	*Clematis jingxiensis* W.T.Wang	2016	IB	77160245-1
	*Clematis maguanensis* W.T.Wang	2015	IB	77147798-1
	*Clematis melanonema* W.T.Wang	2016		77160243-1
	*Clematis wuxiensis* Q.Q.Jiang & H.P.Deng	2017	SW	77160575-1
	*Clematis yuntaishanica* W.T.Wang	2016		77160242-1
	*Delphinium brachyurum* W.T.Wang	2014	SW	77145301-1
	*Delphinium breviscaposum* W.T.Wang	2018	SW	77194230-1
	*Delphinium callichromum* Q.L.Gan & Xin W.Li	2017		77175709-1
	*Delphinium dicentrum* W.T.Wang	2018	H	77193142-1
	*Delphinium filibracteolum* W.T.Wang	2018	SW	77194231-1
	*Delphinium furcatocornutum* W.T.Wang	2014	SW	77137692-1
	*Delphinium lagarocentrum* W.T.Wang	2014		77142465-1
	*Delphinium lagarolobum* W.T.Wang	2018	H	77193145-1
	*Delphinium langxianense* W.T.Wang	2014		77142462-1
	*Delphinium latilimbum* W.T.Wang	2018	H	77193144-1
	*Delphinium longibracteolatum* W.T.Wang	2013	SW	77135649-1
	*Delphinium longziense* W.T.Wang	2018	H	77193139-1
	*Delphinium menyuyanense* W.T.Wang	2016		77160241-1
	*Delphinium pingwuense* W.T.Wang	2015	SW	77147315-1
	*Delphinium quinqueflorum* W.T.Wang	2014	SW	77142464-1
	*Delphinium tephranthum* W.T.Wang	2014	SW	77142463-1
	*Delphinium trichophoroides* W.T.Wang	2014	SW	77142461-1
	*Delphinium viridiovarium* W.T.Wang	2018		77193141-1
	*Delphinium xanthanthum* W.T.Wang	2018	H	77193140-1
	*Delphinium yongdengense* W.T.Wang	2016	SW	77154492-1
	*Delphinium zhanangense* W.T.Wang	2018		77193143-1
	*Delphinium zuogongense* W.T.Wang	2013	SW	77135650-1
	*Dichocarpum wuchuanense* S.Z.He	2015		77150121-1
	*Ranunculus chongzhouensis* W.T.Wang	2015	SW	77150244-1
	*Ranunculus dayiensis* W.T.Wang	2015	SW	77150241-1
	*Ranunculus decandrus* W.T.Wang	2013	SW	77135658-1
	*Ranunculus duoxionglashanicus* W.T.Wang	2013		77135657-1
	*Ranunculus gongheensis* W.T.Wang	2015		77150245-1
	*Ranunculus laohegouensis* W.T.Wang & S.R.Chen	2015	SW	77151736-1
	*Ranunculus lujiangensis* W.T.Wang	2018		77193105-1
	*Ranunculus shanyangensis* M.R.Luo & L.Zhao	2013		77178536-1
	*Ranunculus tongrenensis* W.T.Wang	2015		77150242-1
	*Ranunculus wutaishanicus* W.T.Wang	2016		77160244-1
	*Ranunculus zhouquensis* W.T.Wang	2015	SW	77150243-1
	*Semiaquilegia guangxiensis* Yan Liu & Y.S.Huang	2017	IB	77160163-1
	*Thalictrum austrotibeticum* Jin Y.Li, L.Xie & L.Q.Li	2015		77147128-1
Ranunculaceae	*Thalictrum brachyandrum* W.T.Wang	2017		77163361-1
	*Thalictrum callianthum* W.T.Wang	2013		77135652-1
	*Thalictrum cuonaense* W.T.Wang	2014	H	77145302-1
	*Thalictrum daguanense* W.T.Wang	2017		77165388-1
	*Thalictrum dingjieense* W.T.Wang	2018	H	77194208-1
	*Thalictrum jilongense* W.T.Wang	2017	H	77165391-1
	*Thalictrum lasiogynum* W.T.Wang	2017	SW	77163362-1
	*Thalictrum latistylum* W.T.Wang	2017	SW	77165389-1
	*Thalictrum minutiflorum* W.T.Wang	2017		77163359-1
	*Thalictrum panzhihuaense* W.T.Wang	2016	SW	77154493-1
	*Thalictrum sexnervisepalum* W.T.Wang	2017	SW	77165390-1
	*Thalictrum spiristylum* W.T.Wang	2017	SW	77165392-1
	*Thalictrum tsaii* W.T.Wang	2018		77194199-1
	*Thalictrum xiaojinense* W.T.Wang	2017	SW	77163363-1
	*Thalictrum xinningense* W.T.Wang	2017		77163360-1
	*Thalictrum yadongense* W.T.Wang	2018	H	77194204-1
	*Thalictrum yuoxiense* W.T.Wang	2014		77146200-1
	*Thalictrum zhadaense* W.T.Wang	2017		77165393-1
Rhamnaceae	*Sageretia liuzhouensis* Yi Yang & H.Sun	2017	IB	77174078-1
Rosaceae	*Argentina songzhuensis* T.Feng & Heng C.Wang	2014	SW	77146009-1
	*Cerasus laoshanensis* D.K.Zang	2017		77163885-1
	*Cerasus xueluoensis* C.H.Nan & X.R.Wang	2013		77130902-1
	*Eriobotrya × daduheensis* H.Z.Zhang ex W.B.Liao, Q.Fan & M.Y.Ding	2015	SW	77147555-1
	*Potentilla jiaozishanensis* Huan C.Wang & Z.R.He	2013		77131196-1
	*Potentilla tuberculifera* J.Z.Dong	2017		77175794-1
	*Prunus nutantiflora* D.G.Zhang & Z.H.Xiang	2018		77193174-1
	*Prunus pananensis* Z.L.Chen, W.J.Chen & X.F.Jin	2013		77124052-1
	*Rosa longshoushanica* L.Q.Zhao & Y.Z.Zhao	2016	SW	77155109-1
	*Rubus pseudoswinhoei* Huan C.Wang & Z.R.He	2016		77155201-1
	*Rubus yingjiangensis* Huan C.Wang	2017	IB	77163881-1
	*Sorbus calcicola* W.B.Liao & W.Guo	2016	IB	77155205-1
	*Sorbus cibagouensis* H.Peng & Z.J.Yin	2017	SW	77174586-1
	*Sorbus dolichofoliolatus* X.F.Gao & Meng Li	2015	SW	77150196-1
	*Sorbus prunifolia* W.B.Liao & H.J.Jing	2016	SW	77154795-1
	*Spiraea × transhimalaica* Businský	2015		77149889-1
	*Spiraea fangii* H.Y.Hu & X.J.He	2016	SW	77156678-1
	*Spiraea lanatissima* Businský	2015	SW	77149886-1
Rubiaceae	*Gardenia reflexisepala* N.H.Xia & X.E.Ye	2016	IB	77154583-1
	*Hedyotis austrosinica* L.Wu & L.H.Yang	2018	IB	77179092-1
	*Hedyotis nanlingensis* R.J.Wang	2015	IB	77146981-1
	*Hedyotis taishanensis* G.T.Wang & R.J.Wang	2018	IB	77190121-1
	*Leptodermis hechiensis* R.J.Wang	2018	IB	77178711-1
	*Mussaenda campanulata* T.T.Duan & D.X.Zhang	2016	IB	77155581-1
	*Mycetia fangii* K.J.Yan & Z.Q.Song	2016	IB	77155089-1
	*Ophiorrhiza gaoligongensis* L.Wu, Hareesh & R.H.Tu	2018	SW	77194408-1
	*Ophiorrhiza guizhouensis* C.D.Yang & G.Q.Gou	2018		60476091-2
	*Ophiorrhiza macrocarpa* L.Wu, Q.R.Liu, Y.H.Tan & Hareesh	2018	IB	77179095-1
	*Rubia austrozhejiangensis* Z.P.Lei, Y.Y.Zhou & R.W.Wang	2013		77130002-1
	*Rubia hangii* L.E Yang & Z.L.Nie	2017	IB	77160030-1
	*Rubia pianmaensis* R.Li & H.Li	2013	SW	77133583-1
	*Rubia urceolata* X.F.Wang & C.H.Wang	2018		77190161-1
	*Spiradiclis coriaceifolia* R.J.Wang	2014	IB	77143408-1
	*Spiradiclis danxiashanensis* R.J.Wang	2015	IB	77146980-1
	*Spiradiclis glabra* L.Wu & Q.R.Liu	2016	IB	77161525-1
	*Spiradiclis glandulosa* L.Wu & Q.R.Liu	2014	IB	77146551-1
	*Spiradiclis jingxiensis* R.J.Wang	2016	IB	77158468-1
Rubiaceae	*Spiradiclis longanensis* R.J.Wang	2015	IB	77148932-1
	*Spiradiclis lui* Yan Liu & L.Wu	2018	IB	77188006-1
	*Spiradiclis pauciflora* L.Wu & Q.R.Liu	2015	IB	77174763-1
	*Spiradiclis pengshuiensis* B.Pan & R.J.Wang	2016	SW	77154905-1
	*Spiradiclis quanzhouensis* J.Liu & W.B.Xu	2017	IB	77179034-1
	*Spiradiclis tonglingensis* R.J.Wang	2014	IB	77143409-1
	*Spiradiclis yangchunensis* R.J.Wang	2016	IB	77155443-1
Salicaceae	*Salix alexeii* Kottaim.	2017	SW	77174640-1
Saxifragaceae	*Chrysosplenium zhangjiajieense* X.L.Yu, Hui Zhou & D.S.Zhou	2016		77158754-1
	*Saxifraga kegangii* D.G.Zhang, Ying Meng & M.H.Zhang	2017		77174074-1
	*Saxifraga luoxiaoensis* W.B.Liao, L.Wang & X.J.Zhang	2018		77179043-1
	*Saxifraga viridipetala* Z.X.Zhang & Gornall	2018	SW	77176502-1
Scrophulariaceae	*Bonnaya sanpabloensis* Y.S.Liang & J.C.Wang	2014	IB	77144208-1
	*Mazus sunhangii* D.G.Zhang & T.Deng	2016		77157495-1
	*Pedicularis milliana* W.B.Yu, D.Z.Li & H.Wang	2018	SW	77185944-1
	*Pedicularis wanghongiae* M.L.Liu & W.B.Yu	2015	SW	77147975-1
	*Pterygiella luzhijiangensis* Huan C.Wang	2017		77179094-1
	*Rehmannia chrysantha* M.H.Li & C.H.Zhang	2016		77155584-1
	*Scrophularia jinii* P.Li	2018		77178920-1
Selaginellaceae	*Selaginella chuweimingii* X.M.Zhou, Z.R.He, Liang Zhang & Li Bing Zhang	2015		77150615-1
	*Selaginella daozhenensis* Li Bing Zhang, Q.W.Sun & Jun H.Zhao	2015		77147115-1
	*Selaginella guihaia* X.C.Zhang	2017	IB	60474541-2
	*Selaginella wangpeishanii* Li Bing Zhang, H.He & Q.W.Sun	2014		77140329-1
Smilacaceae	*Smilax hirtellicaulis* C.Y.Wu & C.Chen ex P.Li	2016	IB	77157761-1
	*Smilax microdontus* Z.S.Sun & C.X.Fu	2015		77147575-1
Solanaceae	*Lycium amarum* Lu Q.Huang	2016		77158466-1
Styracaceae	*Styrax rhytidocarpus* W.Yang & X.L.Yu	2015		77150409-1
Taxaceae	*Taxus calcicola* L.M.Gao & Mich.Möller	2013	IB	77136027-1
Theaceae	*Camellia concinna* Orel & Curry	2015	IB	77151428-1
	*Camellia psilocarpa* X.G.Shi & C.X.Ye	2018	IB	77176063-1
	*Camellia tomentosa* Orel & Curry	2015	IB	77151406-1
	*Eurya makuanica* C.X.Ye & X.G.Shi	2015	IB	77154773-1
	*Eurya pilosa* C.X.Ye & X.G.Shi	2015	IB	77174759-1
Thelypteridaceae	*Cyclogramma costularisora* Ching ex K.H.Shing	2014	IB	77145114-1
Thymelaeaceae	*Daphne ogisui* C.D.Brickell, B.Mathew & Yin Z.Wang	2014	SW	77141997-1
Trilliaceae	*Paris caojianensis* B.Z.Duan & Yu Yu Liu	2017	SW	77177679-1
	*Paris nitida* G.W.Hu, Zhi Wang & Q.F.Wang	2017		77176304-1
	*Paris qiliangiana* H.Li, Jun Yang bis & Y.H.Wang	2017		77177750-1
	*Paris tengchongensis* Y.H.Ji, C.J.Yang & Yu L.Huang	2017	SW	77162987-1
Ulmaceae	*Celtis neglecta* Zi L.Chen & X.F.Jin	2017		77161265-1
	*Ulmus erythrocarpa* W.C.Cheng ex Yi F.Duan & X.R.Wang	2016		77158537-1
	*Ulmus kunmingensis* W.C.Cheng	2013		77137832-1
	*Ulmus multinervis* W.C.Cheng ex Yi F.Duan & X.R.Wang	2016		77158536-1
Urticaceae	*Debregeasia hekouensis* W.T.Wang	2016	IB	77156529-1
	*Elatostema androstachyum* W.T.Wang, A.K.Monro & Y.G.Wei	2013	IB	77134637-1
	*Elatostema anlongense* W.T.Wang	2016		77158908-1
	*Elatostema arcuatipes* W.T.Wang & Y.G.Wei	2014		77141252-1
	*Elatostema atrostriatum* W.T.Wang & Y.G.Wei	2013	IB	77178529-1
	*Elatostema austroyunnanense* W.T.Wang	2014	IB	77141437-1
	*Elatostema baoshanense* W.T.Wang & Z.Y.Wu	2016	SW	77165300-1
	*Elatostema biformibracteolatum* W.T.Wang	2014	IB	77141253-1
	*Elatostema bioppositum* L.D.Duan & Yun Lin	2013	IB	77178535-1
	*Elatostema bomiense* W.T.Wang & Z.Y.Wu	2013	SW	77130899-1
	*Elatostema brunneobracteolatum* W.T.Wang	2014	IB	77141264-1
	*Elatostema brunneostriolatum* W.T.Wang	2014	IB	77141413-1
Urticaceae	*Elatostema caudatoacuminatum* W.T.Wang	2013	IB	77144616-1
	*Elatostema chiwuanum* W.T.Wang	2013	SW	77144627-1
	*Elatostema costatoalatum* W.T.Wang & Z.Y.Wu	2014	IB	77141444-1
	*Elatostema crassicostatum* W.T.Wang	2014	IB	77141441-1
	*Elatostema crassimucronatum* W.T.Wang	2014		77141242-1
	*Elatostema cuipingfengense* W.T.Wang & Z.Y.Wu	2016	IB	77165302-1
	*Elatostema cyrtandrifolioides* W.T.Wang	2014	IB	77141269-1
	*Elatostema daxinense* W.T.Wang & Z.Y.Wu	2013	IB	77130900-1
	*Elatostema dentatocaudatum* W.T.Wang & Z.Y.Wu	2016	SW	77165298-1
	*Elatostema femineocymosum* W.T.Wang	2018	SW	77193107-1
	*Elatostema flexuosicaule* W.T.Wang & Z.Y.Wu	2016	SW	77165307-1
	*Elatostema fulvobracteolatum* W.T.Wang	2013	IB	77144630-1
	*Elatostema furcatibracteum* W.T.Wang	2014	H	77141240-1
	*Elatostema furcatiramosum* W.T.Wang	2014	IB	77141412-1
	*Elatostema globosostigmatum* W.T.Wang & Z.Y.Wu	2016	SW	77165334-1
	*Elatostema gyronanophyllum* W.T.Wang	2018	SW	77193109-1
	*Elatostema heterocladum* W.T.Wang, A.K.Monro & Y.G.Wei	2013	IB	77134638-1
	*Elatostema hygrophilifolium* W.T.Wang	2013	IB	77144609-1
	*Elatostema jingxiense* W.T.Wang & Y.G.Wei	2013	IB	77178530-1
	*Elatostema laevicaule* W.T.Wang, A.K.Monro & Y.G.Wei	2013	IB	77134636-1
	*Elatostema linearicorniculatum* W.T.Wang	2014	IB	77141447-1
	*Elatostema longiciliatum* W.T.Wang	2014	IB	77141426-1
	*Elatostema longicuspe* W.T.Wang & Y.G.Wei	2013		77130004-1
	*Elatostema magni*-auriculatum L.D.Duan & Yun Lin	2015	IB	77178674-1
	*Elatostema melanocarpum* W.T.Wang	2013	IB	77144614-1
	*Elatostema melanocephalum* W.T.Wang	2014		77141442-1
	*Elatostema melanoceras* W.T.Wang	2014	IB	77141427-1
	*Elatostema menghaiense* W.T.Wang	2013	IB	77144626-1
	*Elatostema odontopterum* W.T.Wang	2013	IB	77144607-1
	*Elatostema oligotrichum* W.T.Wang	2017	SW	77174976-1
	*Elatostema ornithorrhynchum* W.T.Wang	2014	IB	77141246-1
	*Elatostema pachycephalum* W.T.Wang	2016	IB	77158909-1
	*Elatostema pallidinerve* W.T.Wang	2014	IB	77141254-1
	*Elatostema pellionioides* W.T.Wang	2014	IB	77141241-1
	*Elatostema petiolare* W.T.Wang	2014	IB	77141425-1
	*Elatostema pingbianense* W.T.Wang	2014	IB	77141257-1
	*Elatostema planinerve* W.T.Wang & Y.G.Wei	2013		77130003-1
	*Elatostema pseudolongipes* W.T.Wang & Y.G.Wei	2014	IB	77141420-1
	*Elatostema pseudonanchuanense* W.T.Wang	2014	IB	77141243-1
	*Elatostema purpureolineolatum* W.T.Wang	2013	IB	77144631-1
	*Elatostema quadribracteatum* W.T.Wang	2014	IB	77141255-1
	*Elatostema quinquetepalum* W.T.Wang	2013	IB	77144611-1
	*Elatostema retrostrigulosoides* W.T.Wang	2014	IB	77141272-1
	*Elatostema ronganense* W.T.Wang & Y.G.Wei	2014	IB	77141458-1
	*Elatostema schizodiscum* W.T.Wang & Y.G.Wei	2013		77178531-1
	*Elatostema septemcostatum* W.T.Wang & Z.Y.Wu	2014	IB	77141263-1
	*Elatostema simaoense* W.T.Wang	2013	IB	77144628-1
	*Elatostema simianshanicum* W.T.Wang	2017	SW	77174977-1
	*Elatostema tiechangense* L.F.Fu, Y.G.Wei & A.K.Monro	2017	IB	77160160-1
	*Elatostema tritepalum* W.T.Wang	2014	IB	77141268-1
	*Elatostema viridibracteolatum* W.T.Wang	2014	IB	77141262-1
	*Elatostema viridicarinatum* W.T.Wang	2017	IB	77162747-1
	*Elatostema viridicostatum* W.T.Wang & Z.Y.Wu	2016	SW	77165304-1
	*Elatostema viridinerve* W.T.Wang	2014	IB	77141271-1
	*Elatostema weii* W.T.Wang	2014	IB	77141430-1
	*Elatostema wenshanense* W.T.Wang	2017	IB	77162745-1
Urticaceae	*Elatostema yongtianianum* W.T.Wang	2013		77144619-1
	*Elatostema zhengyuanum* W.T.Wang	2018	SW	77193110-1
	*Elatostema zhenyuanense* W.T.Wang & Z.Y.Wu	2014	IB	77141445-1
	*Laportea jinganensis* W.T.Wang	2016		77156528-1
	*Laportea lageensis* W.T.Wang	2014	H	77138094-1
	*Metapilea jingxiensis* W.T.Wang	2016	IB	77155150-1
	*Pellionia calcifera* W.T.Wang	2016	IB	77154996-1
	*Pellionia laibinensis* W.T.Wang	2017	IB	77164278-1
	*Pellionia mollissima* W.T.Wang	2014	IB	77139051-1
	*Pellionia simianschanica* W.T.Wang	2017	SW	77174975-1
	*Pellionia tritepala* W.T.Wang	2016		77154995-1
	*Pilea gongjueensis* W.T.Wang	2016		77156533-1
	*Pilea lageensis* W.T.Wang	2014	H	77138095-1
	*Pilea longruiensis* W.T.Wang	2017	IB	77174980-1
	*Pilea longzhouensis* W.T.Wang	2017	IB	77174978-1
	*Pilea luochengensis* W.T.Wang	2016	IB	77156532-1
	*Pilea lushuiensis* W.T.Wang	2017	SW	77174979-1
	*Pilea minima* W.T.Wang	2017	SW	77174981-1
	*Pilea nonggangensis* Y.G.Wei, L.F.Fu & A.K.Monro	2017	IB	77176278-1
	*Pilea weimingii* Huan C.Wang	2018		77186306-1
	*Pilea yuanbaoshanica* W.T.Wang	2017	IB	77174983-1
	*Urtica chengkouensis* W.T.Wang	2017	SW	77164277-1
	*Urtica malipoensis* W.T.Wang	2014	IB	77140819-1
	*Zhengyia shennongensis* T.Deng, D.G.Zhang & H.Sun	2013		77125828-1
Violaceae	*Viola hybanthoides* W.B.Liao & Q.Fan	2015	IB	77145097-1
	*Viola nujiangensis* Y.S.Chen & X.H.Jin	2015	SW	77150548-1
Vitaceae	*Cyphostemma dehongense* L.M.Lu & V.C.Dang	2017	IB	77167306-1
	*Pseudocayratia speciosa* J.Wen & L.M.Lu	2018	IB	77193893-1
Woodsiaceae	*Athyrium sessilipinnum* X.C.Zhang & R.Wei	2016	IB	77160655-1
	*Diplazium yinchanianum* Zi Yue Liu, H.J.Wei & Y.H.Yan	2018	IB	77177560-1
	*Hypodematium confertivillosum* J.X.Li, F.Q.Zhou & X.J.Li	2018		77174973-1
Zingiberaceae	*Amomum hainanense* Y.S.Ye, J.P.Liao & P.Zou	2018	IB	77193005-1
	*Amomum velutinum* X.E.Ye, Škorničk. & N.H.Xia	2017	IB	77187998-1
	*Boesenbergia kingii* Mood & L.M.Prince	2013	IB	77130879-1
	*Curcuma gulinqingensis* N.H.Xia & Juan Chen	2013	IB	77135581-1
	*Hedychium dichotomatum* Picheans. & Wongsuwan	2013	IB	77127653-1
	*Hedychium viridibracteatum* X.Hu	2018	IB	77191580-1
	*Roscoea glaucifolia* F.J.Mou	2015	SW	77149685-1
	*Zingiber hainanense* Y.S.Ye, L.Bai & N.H.Xia	2015	IB	77147977-1
	*Zingiber leucochilum* L.Bai, Škorničk. & N.H.Xia	2018	SW	77192884-1
	*Zingiber pauciflorum* L.Bai, Škorničk., D.Z.Li & N.H.Xia	2017	IB	77179030-1
	*Zingiber tenuifolium* L.Bai, Škorničk. & N.H.Xia	2015	IB	77150125-1
	*Zingiber ventricosum* L.Bai, Škorničk., N.H.Xia & Y.S.Ye	2016	IB	77155186-1
	*Zingiber zhuxiense* G.X.Hu & S.Huang	2015		77174765-1

*Biodiversity hotspots abbreviation. CA: Mountains of Central Asia, H: Himalaya, IB: Indo-Burma, SW: Mountains of Southwest China, Blank: non-biodiversity hotspots area. Species which recognized as nomen illegitimum were excluded.
